# Retinylidene chromophore hydrolysis from mammalian visual and non-visual opsins

**DOI:** 10.1016/j.jbc.2024.105678

**Published:** 2024-01-23

**Authors:** John D. Hong, David Salom, Elliot H. Choi, Samuel W. Du, Aleksander Tworak, Roman Smidak, Fangyuan Gao, Yasmeen J. Solano, Jianye Zhang, Philip D. Kiser, Krzysztof Palczewski

**Affiliations:** 1 Department of Ophthalmology, Gavin Herbert Eye Institute, University of California Irvine, Irvine, California, USA; 2 Department of Chemistry, University of California Irvine, Irvine, California, USA; 3 Department of Physiology and Biophysics, University of California Irvine, Irvine, California, USA; 4 Department of Clinical Pharmacy Practice, University of California Irvine, Irvine, California, USA; 5 Research Service, VA Long Beach Healthcare System, Long Beach, California, USA; 6 Department of Molecular Biology and Biochemistry, University of California Irvine, Irvine, California, USA

**Keywords:** 11-*cis*-retinal, all-*trans*-retinal, chromophore, retinal hydrolysis, visual cycle

## Abstract

Rhodopsin (Rho) and cone opsins are essential for detection of light. They respond *via* photoisomerization, converting their Schiff-base-adducted 11-*cis*-retinylidene chromophores to the all-*trans* configuration, eliciting conformational changes to activate opsin signaling. Subsequent Schiff-base hydrolysis releases all-*trans*-retinal, initiating two important cycles that maintain continuous vision—the Rho photocycle and visual cycle pathway. Schiff-base hydrolysis has been thoroughly studied with photoactivated Rho but not with cone opsins. Using established methodology, we directly measured the formation of Schiff-base between retinal chromophores with mammalian visual and nonvisual opsins of the eye. Next, we determined the rate of light-induced chromophore hydrolysis. We found that retinal hydrolysis from photoactivated cone opsins was markedly faster than from photoactivated Rho. Bovine retinal G protein-coupled receptor (bRGR) displayed rapid hydrolysis of its 11-*cis*-retinylidene photoproduct to quickly supply 11-*cis*-retinal and re-bind all-*trans*-retinal. Hydrolysis within bRGR in native retinal pigment epithelium microsomal membranes was >6-times faster than that of bRGR purified in detergent micelles. N-terminal-targeted antibodies significantly slowed bRGR hydrolysis, while C-terminal antibodies had no effect. Our study highlights the much faster photocycle of cone opsins relative to Rho and the crucial role of RGR in chromophore recycling in daylight. By contrast, in our experimental conditions, bovine peropsin did not form pigment in the presence of all-*trans*-retinal nor with any mono-*cis* retinal isomers, leaving uncertain the role of this opsin as a light sensor.

Vision begins with the photochemistry of the opsin-chromophore pigment complexes that reside in the photoreceptor outer segments. Incident light induces photoisomerization of the adducted 11-*cis*-retinylidene chromophore to produce the all-*trans*-retinylidene agonist. Photoproduction of the agonist immediately leads to conformational changes in opsin, which converts to its photoactivated signaling state and initiates the phototransduction cascade (1, 2). The subsequent hydrolysis of the Schiff-base-bound all-*trans* agonist leads to its release from opsin as all-*trans*-retinal (3). This bleachable property of these pigments is characteristic of rhodopsin (Rho) and cone opsins responsible for vertebrate vision. The process of photoisomerization and hydrolysis (bleaching) supplies all-*trans*-retinal to the visual cycle to be recycled to 11-*cis*-retinal to maintain the photocycle of the opsins (4). Notably, cone opsins have been understood to bleach significantly faster than Rho, based on indirect spectroscopic and electrophysiological measurements (5, 6, 7). However, there has yet to be any direct measurements of the bleaching rate of cone pigments, as has been done recently for Rho (8, 9).

Recycling of 11-*cis*-retinal from all-*trans*-retinal can be driven either by a light-dependent process through retinal G protein-coupled receptor (RGR) (10) or by the light-independent classical visual cycle (11). Given the slow rate of dark adaptation, the classical visual cycle pathway of 11-*cis*-retinal production would be insufficient under daylight conditions to maintain high light sensitivity (12, 13); thus, RGR provides a critical, alternative route using light to ultimately supply 11-*cis*-retinal (10, 14). Recently, the rate of the hydrolytic release of 11-*cis*-retinal from RGR after photoisomerization of its all-*trans*-retinylidene adduct was found to be exceedingly fast at roughly 60-times the rate of hydrolytic release of all-*trans* agonist from photoactivated Rho (15). The structural features of RGR that modulate the hydrolysis of 11-*cis*-retinylidene photoproduct have yet to be discovered.

Notably, the photoisomerization of the 11-*cis*- to all-*trans*-retinylidene is far more common among vertebrate opsins as compared to the *trans-cis* isomerization exhibited by RGR. Like RGR, vertebrate peropsin (RRH) also resides in the retinal pigment epithelium (RPE), specifically in the apical surface (16). RRH was also reported to be potentially capable of the *trans-cis* isomerization; however, its pigment formation and isomerization has yet to be characterized, since most studies have focused on an invertebrate homolog (17, 18).

Previously, photoisomerization of the 11-*cis*-retinylidene chromophore of Rho to the all-*trans*-retinylidene agonist was captured in membrane preparations of rod outer segments (ROSs) and in detergent micelles and quantified by liquid chromatography with tandem mass spectrometry (LC-MS/MS) analysis (9). The same analytical procedure tracked the hydrolytic release of the Schiff-base–bound agonist from Rho in either milieu. Sample preparation using NaBH_4_ in isopropanol (NaBH_4_/*i*PrOH) facilitated the reductive trapping of the opsin-bound chromophore and isolation of the opsin protein *via* precipitation; then, proteolysis and LC-MS/MS analysis identified and quantified the products, as illustrated schematically in Figure 1. The photoisomerization reaction of any opsin that forms pigment, as well as the hydrolytic release of the Schiff-base-bound photoproduct of the chromophore, can be studied *via* this technique.

**Figure 1:**
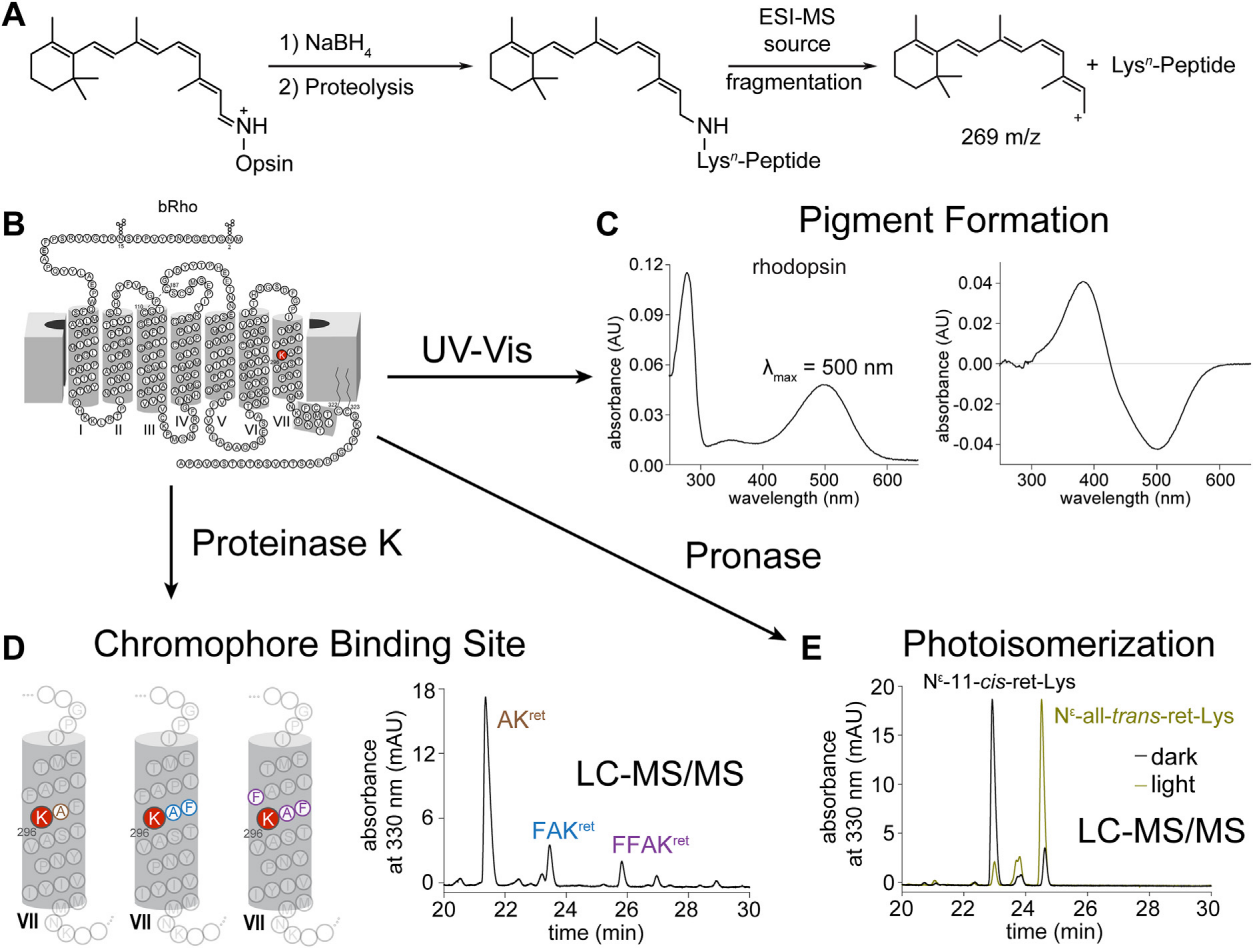
**Schematic diagram of the protocol for determining retinylidene-opsin pigment chromophore-binding site and photoisomerization reaction.***A*, overall scheme of sample preparation for MS analysis. *B*, opsins are mapped to determine presence of an internal Lys residue for retinylidene Schiff base attachment within the putative chromophore binding pocket. *C*, the formation of retinylidene-opsin pigment, as well as the shift in the absorption maximum following exposure to light, is characterized by UV-Vis spectrophotometry. *D*, retinylidene-opsin pigments are treated with NaBH_4_ in isopropanol to reductive trapping of chromophore. After proteolysis by proteinase K, LC-MS/MS analyses can detect resultant N^ε^-retinyl peptides, which fragment during electrospray ionization (ESI) to a retinyl cation and product peptide ion, which can be sequenced by collision-induced dissociation fragmentation. *E*, the photoisomerization of the chromophore is documented by LC-MS/MS analysis of the pronase digest of the pre-illuminated and postilluminated opsin pigment treated with NaBH_4_. Analyses at various time points after illumination can be performed to monitor hydrolysis kinetics of the chromophore photoproduct.

Our current study aimed to directly measure the relative bleaching rate of cone opsins compared to Rho, utilizing LC-MS based methods. Here, these same approaches are applied to bRGR with various structural manipulations to interrogate features that impact its hydrolytic release of 11-*cis*-retinal. Furthermore, we investigate whether bRRH behaves similarly to bRGR in forming pigment with all-*trans*-retinal for subsequent *trans-cis* photoisomerization.

## Results

### Proteolysis of borohydride-reduced visual pigments and LC-MS/MS analysis of resultant N^ε^-retinyl-peptides

**Figure 2:**
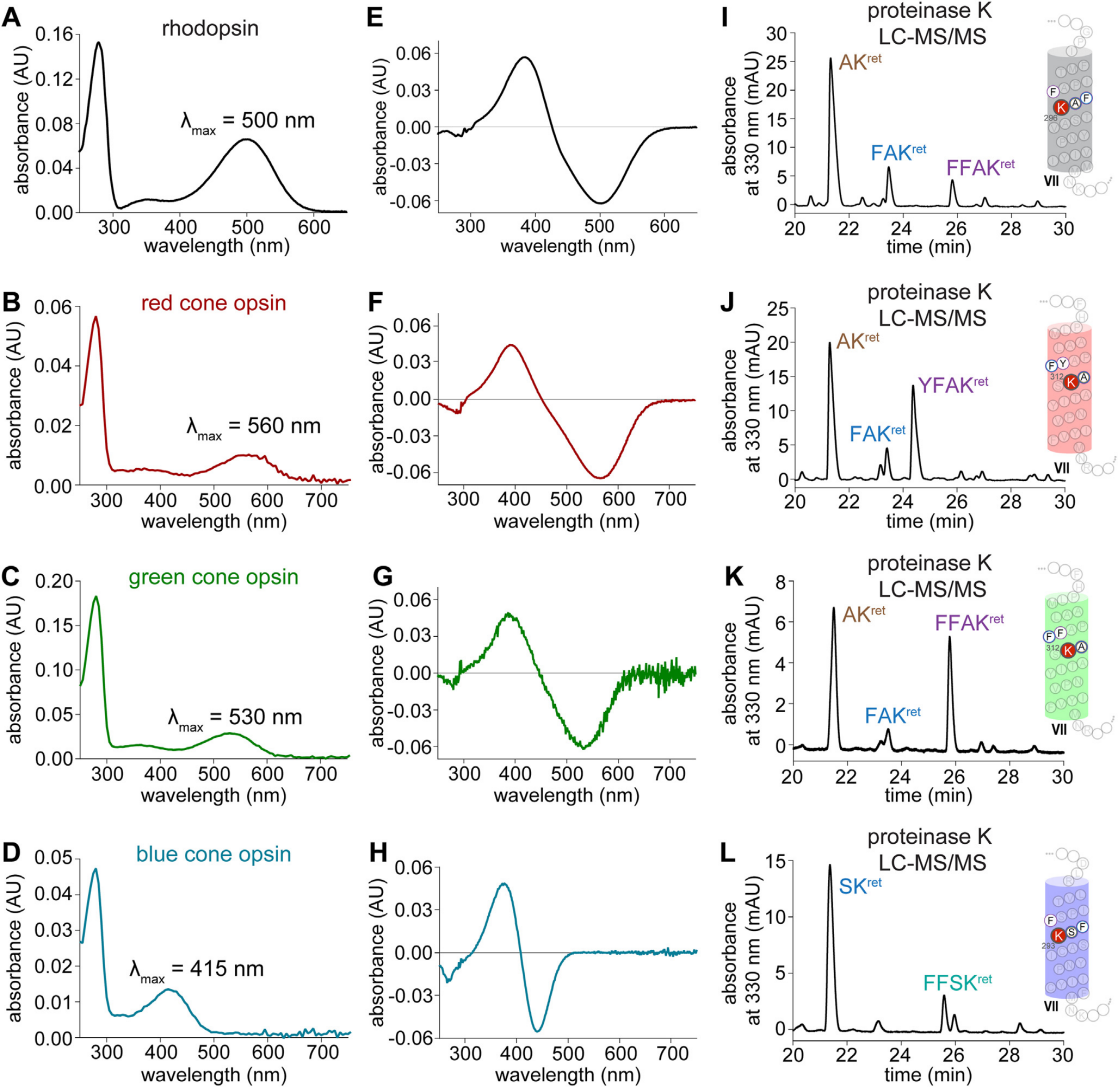
**Spectral characterization and chromophore-binding site identification of visual pigments.***A*–*D*, UV-Vis spectrum of purified bRho (panel *A*), hRed-cone opsin (panel *B*), hGreen-cone opsin (panel *C*), and hBlue-cone opsin (panel *D*) in LMNG. *E*–*H*, characteristic difference spectra after illumination of purified bRho (panel *E*), hRed (panel *F*), hGreen (panel *G*), and hBlue (panel *H*) in LMNG*. I*–*L*, chromatographic separation and MS/MS identification of N^ε^-retinyl-peptides from the proteinase K digest of NaBH_4_/*i*PrOH-treated purified bRho (panel *I*), hRed (panel *J*), hGreen (panel *K*), and hBlue (panel *L*). Location of the chromophore-binding residue within helix VII is identified by the corresponding labeled N^ε^-retinyl-peptide fragments detected from the proteinase K digest of each opsin-chromophore preparation. Detailed LC-MS/MS analyses are shown in Figs. S1–S5. *i*PrOH, isopropanol; LMNG, lauryl maltose neopentyl glycol.

The principal visual pigments, namely Rho and the cone opsins, were obtained from bovine ROS and from recombinant expression, respectively. Pigment formation with each visual opsin was confirmed by UV-Vis absorbance spectroscopy (Fig. 2). Following treatment with NaBH_4_/*i*PrOH, each opsin was proteolyzed by proteinase K to produce N^ε^-retinyl-peptides. Subsequent mass spectral analyses confirmed the location of the chromophore bound to the expected internal Lys-residue within the chromophore-binding pocket of the respective opsins (Figs. S1–S5).

The procedure was repeated using NaBD_4_ in place of NaBH_4_, which verified the reductive trapping of the chromophore-Schiff base-attachment by deuterium labeling of the C^15^ position of the resultant retinyl group, consequently in secondary amine linkage to opsin (Figs. S1–S5). Use of NaBD_4_ for reductive trapping of chromophore and LC-MS/MS analysis of the proteolysis products also later confirmed the characteristic fragmentation pattern of retinyl-peptides into a product retinyl cation and a product-free peptide ion, which can be sequenced by collision-induced dissociation fragmentation. For NaBH_4_, the retinyl cation had an expected m/z of 269, while the NaBD_4_ treatment resulted in a retinyl cation with an expected m/z of 270.

**Figure 3:**
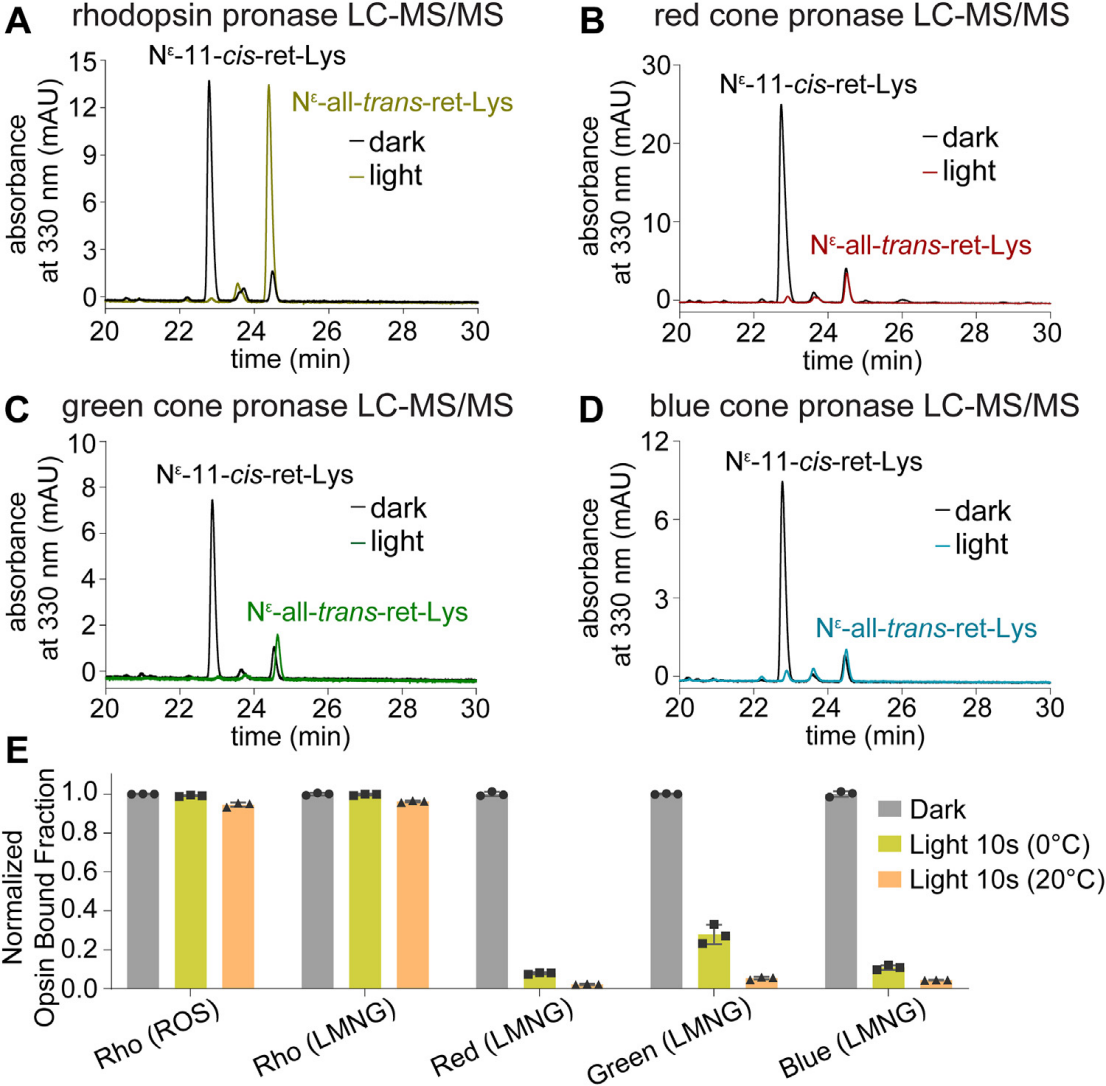
**Characterization of visual pigment photoisomerization reaction and subsequent hydrolysis of all-*trans* agonist.***A*–*D*, chromatographic separation of N^ε^-retinyl-Lys products from pronase digests of photoactivated and ground-state bRho (panel *A*), hRed-cone opsin (panel *B*), hGreen-cone opsin (panel *C*), and hBlue-cone opsin (panel *D*), each treated with NaBH_4_/*i*PrOH. Chromatograms in panels (*A*–*D*) reflect the chromophore photoisomerization observed at 0 °C. Detailed LC-MS/MS analyses are shown in Figs. S6–S10. *E*, comparison of the extents of hydrolysis of the all-*trans*-retinylidene agonists, demonstrating much faster hydrolysis of the agonist in photoactivated cones opsins than in photoactivated rhodopsin. Each bar represents the mean from three replicates, represented as data points, and the error bars represent the standard deviation. *i*PrOH, isopropanol; LMNG, lauryl maltose neopentyl glycol; ROS, rod outer segment.

The use of pronase in place of proteinase K allowed for complete proteolysis and detection of relevant isomers (11-*cis* or all-*trans*) of N^ε^-retinyl-Lys. For pigments treated with NaBH_4_ or NaBD_4_ in the dark, the resultant N^ε^-retinyl-Lys was predominantly in the 11-*cis* configuration; however, upon brief illumination of pigments, the 11-*cis* peak decreased with emergence of a dominant all-*trans* peak (Figs. 3, S6, S7, S8, S9, and S10). Although UV-Vis spectroscopy also detects this *cis*-*trans* photochemistry with the shift in absorbance spectrum of each pigment to the spectrum of the photoactivated state (λ_max_ = 380 nm), subsequent Schiff-base hydrolysis of the all-*trans* agonist cannot be properly resolved due to the overlapping spectrums of photoactivated opsin and all-*trans*-retinal, each with λ_max_ = 380 nm. Since LC-MS/MS of the pronase proteolysis detects bound retinal as N^ε^-retinyl-Lys, the extent of Schiff-base hydrolysis of the all-*trans* agonist in photoactivated cone opsins and Rho can be compared. During the brief illumination of cone opsins, most of the N^ε^-all-*trans*-retinyl-Lys peak had already disappeared, with only 3 to 5% bound agonist remaining in photoactivated cone opsins, in contrast to the 95% remaining in photoactivated Rho at 20 °C (Fig. 3).

### Proteolysis and LC-MS/MS analysis of bRGR photoisomerization reaction and production of 11-*cis*-retinal

**Figure 4:**
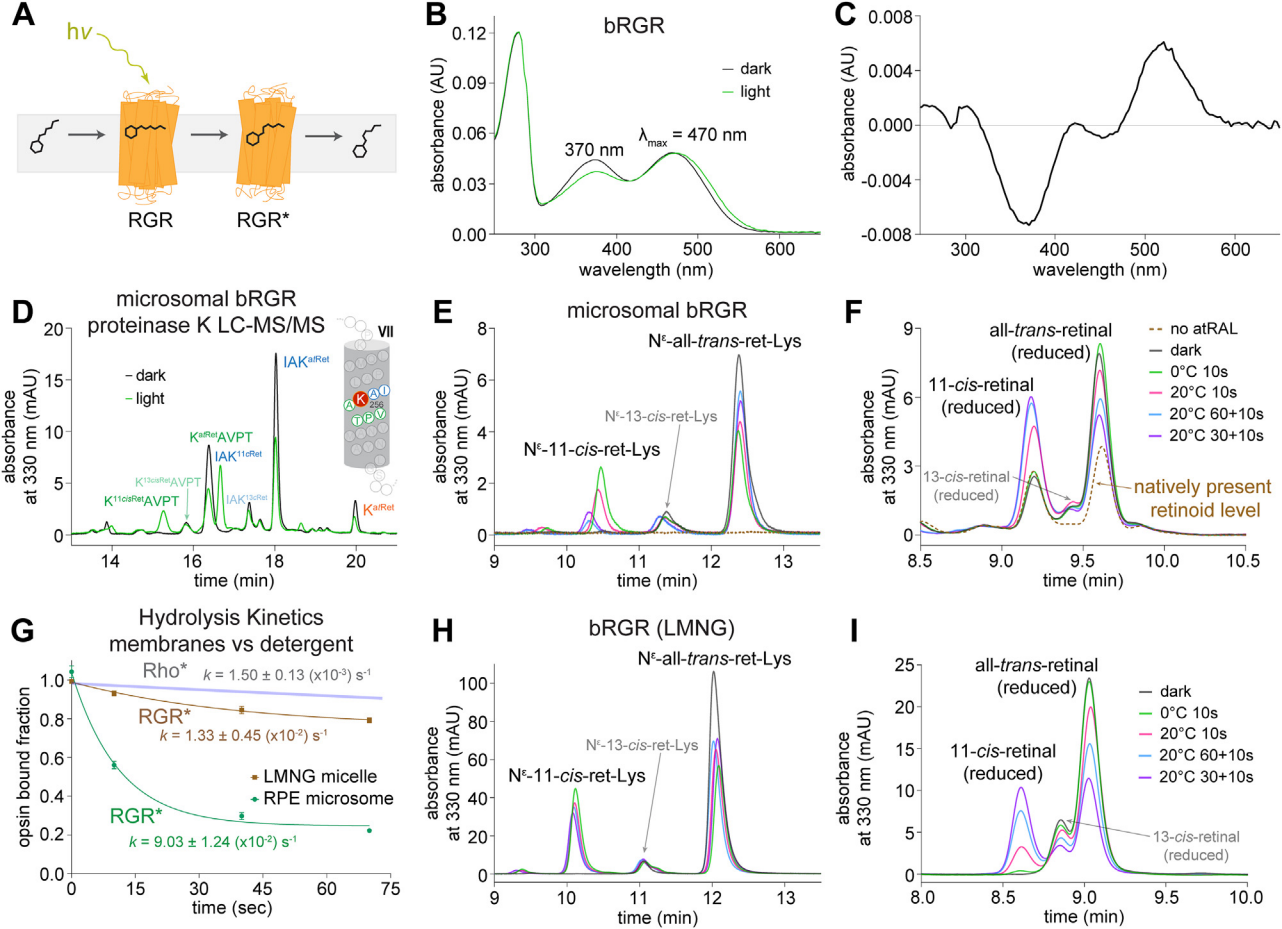
**LC-MS/MS analysis of the bRGR pigment photoisomerization reaction and subsequent photoproduct hydrolysis.***A*, schematic diagram of bRGR photoconversion of all-*trans*-retinal to 11-*cis*-retinal in bRPE microsomal membranes. *B*, absorbance spectrum before and after illumination of recombinant bRGR reconstituted with all-*trans*-retinal and purified in LMNG. *C*, difference spectrum after illumination of bRGR, showing relatively minimal changes. *D*, chromatographic separation of N^ε^-retinyl-peptide products from the proteinase K digest of microsomal bRGR, before and after light exposure, with subsequent NaBH_4_/*i*PrOH treatment. Location of chromophore-binding residue within helix VII identified as labeled N^ε^-retinyl-peptide fragments detected from the proteinase K digest. Detailed LC-MS/MS analyses are shown in Figs. S12–S15. *E* and *H*, chromatographic detection of *trans*-*cis* bRGR photoisomerization as well as subsequent hydrolysis of the 11-*cis*-retinylidene adduct, with a corresponding increase in new all-*trans*-retinylidene adduct formation. *F* and *I*, reciprocal increase in 11-*cis*-retinal in the supernatant produced by hydrolysis and decrease in all-*trans*-retinal, with new bRGR pigment formation. *G*, hydrolysis kinetics: the hydrolysis of the 11-*cis*-retinylidene photoproduct was dramatically slower by nearly 6-fold for bRGR in detergent micelles in contrast to bRGR in native RPE microsomal membranes. The hydrolysis was still faster in either case than the hydrolysis of the all-*trans* agonist from photoactivated rhodopsin, whose hydrolysis plot was adapted from Hong *et.al.* (9). bRGR, bovine RGR; bRPE, bovine RPE; LMNG, lauryl maltose neopentyl glycol; RGR, retinal G protein-coupled receptor; RPE, retinal pigment epithelium.

The photic recycling of all-*trans*-retinal to 11-*cis*-retinal is facilitated by bRGR in two steps: photoisomerization of the all-*trans*-retinylidene adduct to the 11-*cis* adduct, which then must be hydrolyzed to release 11-*cis*-retinal for regeneration of visual opsin pigments (Fig. 4*A*). Following photoisomerization, occurring on the timescale of femtoseconds, the hydrolysis of the bRGR photoproduct (bRGR∗) was recently determined to occur rapidly in native bovine RPE (bRPE) microsomal membranes with a half-life of around 7.5 s (15). For a better understanding of purified bRGR, a mammalian cell line was establishing in HEK293S GnTI^-^ with stable expression of bRGR C-terminally tagged with 1D4 peptide for eventual immunopurification (Fig. S11). The recombinantly produced bRGR adducted with all-*trans*-retinal was immunopurified in lauryl maltose neopentyl glycol (LMNG) micelles for measurement of the bRGR. UV-Vis absorbance spectrum (Figs. 4*B*, S12, and S13). The site of chromophore attachment to Lys^256^ in bRGR was verified by NaBH_4_/D_4_ treatment followed by proteolysis by proteinase K and LC-MS/MS analyses (Fig. S12). Following brief illumination, changes in the UV-Vis absorbance spectrum were found to be minor (Fig. 4, *B* and *C*) and were unable to clearly depict that photoisomerization of the all-*trans*-retinylidene adduct to the 11-*cis*-configuration had occurred. The photoisomerization reaction of bRGR was captured by LC-MS/MS of the pronase digestion of bRGR before and after light exposure, treated with NaBH_4_/D_4_ (Fig. S13).

In parallel to the photoisomerization detailed for the recombinant bRGR, the photoisomerization reaction of native bRGR in isolated bRPE microsomal membranes was also investigated. Microsomal bRGR reconstituted with slight excess of all-*trans*-retinal was analyzed by LC-MS/MS after NaBH_4_ treatment and proteinase K digestion. The resultant N^ε^-retinyl-peptides were determined to be from bRGR with Lys^256^ as the site of chromophore attachment. The photoisomerization reaction of bRGR within the complex environment of bRPE microsomes was validated by LC-MS/MS analysis of the proteinase K (Fig. 4*D*) and pronase digests (Fig. 4*E*), before and after exposure of the all-*trans*-retinal–treated microsomes to light. The rapid hydrolytic production of 11-*cis*-retinal by bRGR in native membranes was recapitulated in this study (Fig. 4, *D*–*G*) with a half-life of around 7.7 s. However, this hydrolysis rate was dramatically slowed by nearly 6-fold for bRGR depleted of cell membrane lipids *via* immunopurification in detergent micelles (Fig. 4, G–I). Notably, the hydrolysis of the bRGR photoproduct in detergent micelles was still faster than hydrolysis of agonist from photoactivated Rho in native membranes. This observation highlights the rapid speed at which bRGR supplies 11-*cis*-retinal following reverse photoisomerization.

### Modulation of the bRGR photoproduct hydrolysis by antibodies

**Figure 5:**
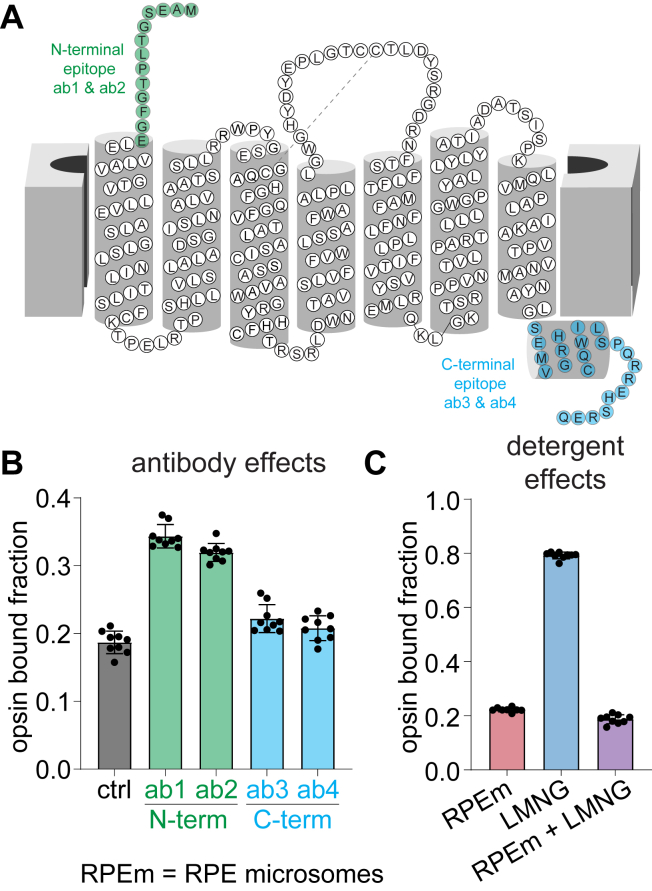
**Attenuation of bRGR photoproduct hydrolysis by anti-N-terminal antibodies.***A*, epitope mapping displaying targeting of a pair of monoclonal anti-bRGR antibodies to the N terminus (*green*) and another pair to the C terminus (*blue*). *B*, after 1 min following a 10-s illumination with 530-nm light of bRPE microsomes solubilized in 1% w/v LMNG, the anti-N-terminal antibodies slowed the extent of hydrolysis of the bRGR photoproduct, whereas the anti-C-terminal antibodies showed minor effect on hydrolysis. *C*, the extent of hydrolysis of the 11-*cis*-retinylidene adduct was nearly unperturbed by the introduction of 1% w/v LMNG detergent to bRPE microsomes; however, the depletion of membrane lipids with immunopurification of bRGR in LMNG micelles dramatically slowed hydrolysis. For panels (*B*) and (*C*), points represent individual replicates (n = 9), bars represent mean values, and error bars represent standard deviation. bRGR, bovine RGR; bRPE, bovine RPE; LMNG, lauryl maltose neopentyl glycol; RGR, retinal G protein-coupled receptor; RPE, retinal pigment epithelium.

Monoclonal bRGR antibodies were produced from hybridoma cultures generated from murine B-cells immunized with bRGR protein (Fig. S16). To study their effect on hydrolysis of the 11-*cis*-retinylidene photoproduct of bRGR, two anti-N-terminal antibodies and two anti-C-terminal antibodies (Fig. 5*A*) were incubated with bRGR in bRPE microsomes, which were solubilized in 1% w/v LMNG to allow free access of antibodies to either side of the bRGR opsin protein. At 1 min postillumination, the extent of hydrolytic release of the 11-*cis*-retinal photoproduct was about 67% in the presence of either of the N-terminal antibodies, compared to 81% in the absence of antibodies and 79% in the presence of either of the C-terminal antibodies (Fig. 5*B*). Notably, the addition of LMNG at 1% w/v to solubilize RPE microsomal membranes appeared to minimally affect the extent of hydrolysis, compared to native membranes alone (Fig. 5*C*); however, the depletion of phospholipids by 1D4 immunoaffinity purification of bRGR significantly slowed the hydrolysis (Fig. 5*C*), as described above.

### Thermostability study of bRho and bRGR

**Figure 6:**
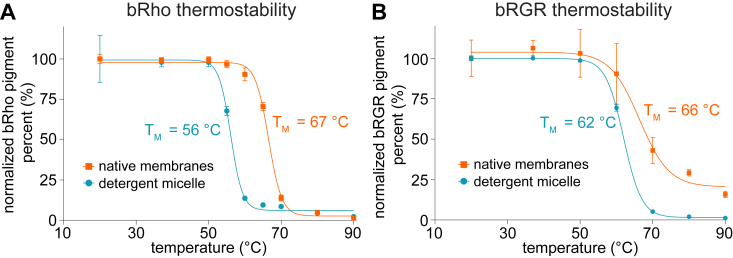
**Reduction of bRho and bRGR in detergent micelles depleted of native lipids.***A*, the high thermostability of bRho in native ROS membranes is significantly diminished upon solubilization in LMNG detergent micelles and depletion of native lipids, with a decrease in T_m_ from 67 °C to 56 °C. *B*, similar findings were observed for bRGR in native bRPE microsomal membranes, with a high T_m_ = 66 °C which decreased to 62 °C for bRGR in LMNG detergent micelles depleted of lipids. For panels (*A*) and (*B*), points mean values, and error bars represent standard deviation with n = 6. bRGR, bovine RGR; LMNG, lauryl maltose neopentyl glycol; RGR, retinal G protein-coupled receptor; ROS, rod outer segment.

Prior studies on the thermostability of bRho in detergent micelles showed thermal isomerization and release of chromophore at 60 °C (19, 20, 21). Native membranes confer significant thermostability to the bRho pigment as shown by the significantly higher melting temperature (T_m_) of 67 °C in bovine ROS membranes as compared to 56 °C in detergent micelles depleted of membrane lipids (Fig. 6*A*). The T_m_ of bRGR pigment was close to that of bRho pigment in native membranes with a T_m_ of 66 °C. In detergent micelles depleted of membrane lipids, the T_m_ of bRGR pigment was reduced to 62 °C, indicating a common effect of lipids in stabilizing visual-associated opsins. (Fig. 6*B*).

### Mammalian RRH is a nonpigment forming opsin

Prior work with invertebrate RRH suggested a role similar to RGR in the photic production of 11-*cis*-retinal from all-*trans*-retinal (17, 18). Therefore, this potential analogous role was explored with mammalian RRH, testing bRRH in the same manner as for bRGR (above). As shown in Table 1, prior single-cell RNA-sequencing data for human, mouse, and bovine RPE cells revealed a similar pattern of expressions of visual cycle elements (10, 22, 23, 24). The expression of bRRH appears to be significantly lower than other elements in the RPE, except for mouse RPE where the expression of RRH is significantly higher compared to human or bovine RPE, aligning with prior studies investigating the presence of mouse RRH in the RPE apical surface (16, 25).

**Table 1:** Single cell RNA sequencing and proteomics of various visual cycle components in the RPE^a^

Gene	Human RPE	Mouse RPE	Bovine RPE	Bovine RPE (lysis buffer 1)^b^^,^^c^	Bovine RPE (lysis buffer 2)^b^^,^^d^	Bovine RPE (lysis buffer 3)^c^^,^^e^
	scRNAseq^a^			Proteomics^a^		
RRH	0.6	4.2	0.3	0.19	0.0	0.039
CRALBP (RLBP1)	34.5	24.8	74.0	3.0	4.7	0.84
IRBP (RBP3)	5.3	2.1	0.9	4.2	4.2	4.0
RGR	25.1	111.8	49.9	4.1	7.0	1.8
LRAT	4.5	7.8	11.9	0.43	2.6	0.0
RPE65	114.4	42.1	61.3	5.6	22.4	4.2
RDH5	2.2	53.6	8.1	2.0	12.1	1.4
RDH10	6.7	14.6	0.3	0.15	0.27	0.0

Abbreviations: CRALBP, cellular retinaldehyde-binding protein; IRBP, interphotoreceptor retinoid-binding protein; LRAT, lecithin retinol acyltransferase; RDH, retinol dehydrogenase; RLBP, retinaldehyde-binding protein; RPE65, retinoid isomerase; RRH, peropsin.

a The scRNAseq were generated in previous studies (10, 22, 23, 24). Proteomic data are obtained in this work as described in the Supplemental Method section. For scRNAseq, the data represent the relative average expression values calculated for the RPE cell clusters using the average expression function of Seurat; for proteomics, the data are expressed in intensity-based absolute quantification (iBAQ) units.

b iBAQ values were determined by the sum of ion intensities (×10^7^) of peptides matching a specific protein divided by the theoretical number of observable tryptic peptides.

c Buffer:10 mM Tris/HCl, pH 8.3, containing 0.1% SDS.

d Buffer:10 mM Tris/HCl, pH 8.3, containing 4% SDS.

e Buffer:10 mM Tris/HCl, pH 8.3, containing 8 M urea.

**Figure 7:**
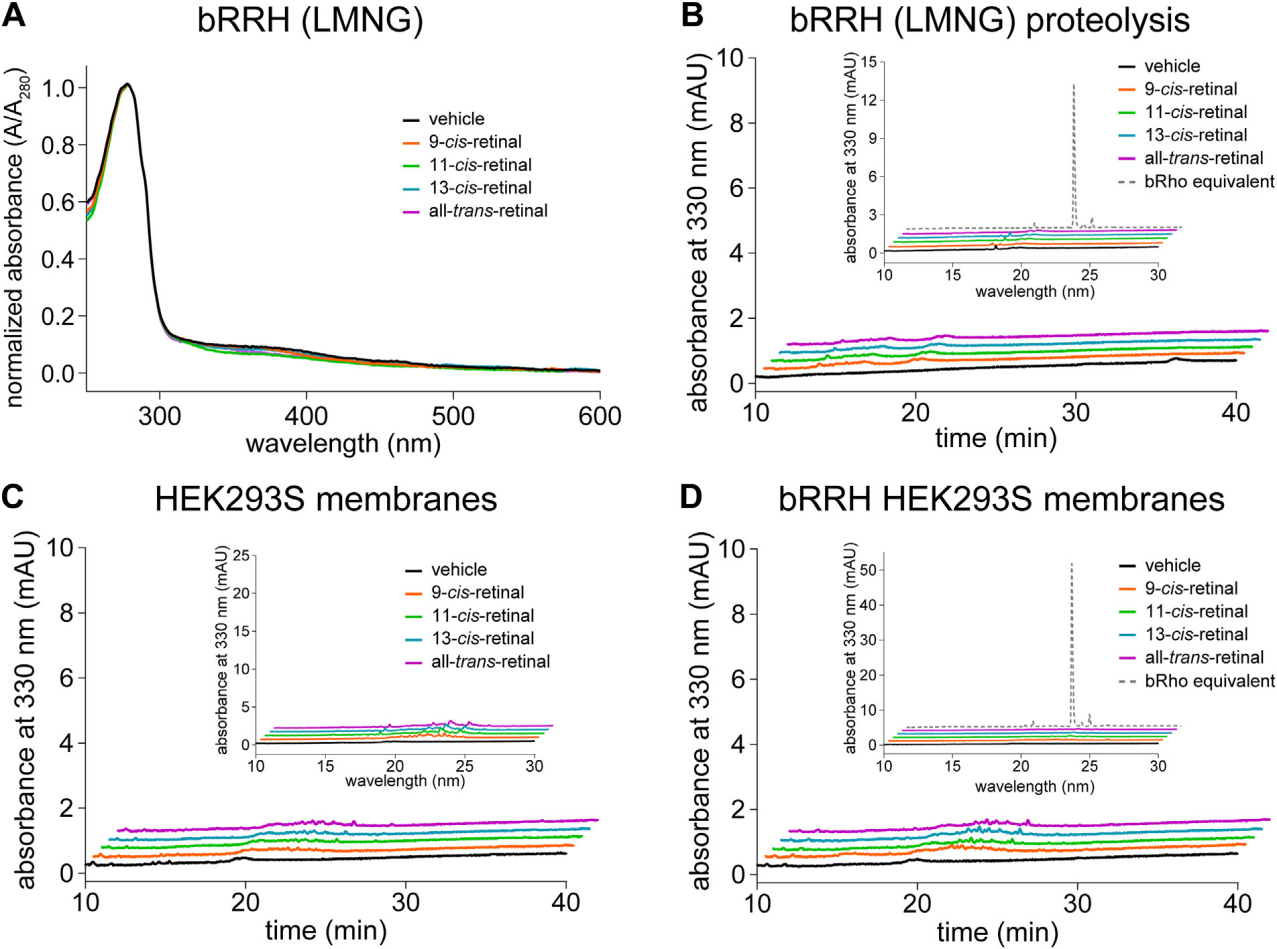
**Lack of pigment formation by bRRH treated with retinal.***A*, absorbance spectra of RRH treated with different retinal isomers and subsequently purified in LMNG showed no difference from vehicle treatment, indicating lack of pigment formation with retinal. *B*, LC-MS/MS analysis of the proteinase K digest of NaBH_4_-reduced immunopurified RRH that had been treated with retinal isomers did not detect N^ε^-retinyl-peptides nor did LC-MS/MS analysis of the corresponding pronase digest detect any N^ε^-retinyl-Lys (inset). LC-MS/MS of the pronase digest of an amount of bRho equivalent to bRRH was performed to display the amount of N^ε^-retinyl-Lys signal that would be expected if retinylidene-Schiff base adducted bRRH were present. *C*, the same LC-MS/MS analyses of the proteinase K and pronase digests (inset) were done for membrane isolates of HEK293S cells treated with retinal isomers, showing no definitive N^ε^-retinyl-peptides and N^ε^-retinyl-Lys, respectively. *D*, the same protocol was repeated with membrane isolates of HEK293S cells stably expressing bRRH, also showing lack of any definitive signals of N^ε^-retinyl-peptides or N^ε^-retinyl-Lys. LMNG, lauryl maltose neopentyl glycol; RRH, peropsin.

Given the low abundance of bRRH from native bRPE sources (Table 1), a mammalian cell line was established in HEK293S GnTI^-^ with stable expression of bRRH (Fig. S17). GFP was localized to the cytoplasm, whereas ID4-tagged bRRH was present in the plasma membranes. Membrane isolates of bRRH were treated with different retinal isomers and subsequently immunopurified in LMNG detergent for facile UV/Vis spectroscopic detection of any potential bRRH pigments. However, the absorbance spectra of bRRH treated with each retinal isomer showed negligible difference from that of bRRH treated with vehicle (Fig. 7*A*), suggesting a lack of pigment formation. Subsequent borohydride treatment and proteolysis by either proteinase K or pronase was performed for LC-MS/MS detection of any N^ε^-retinyl-peptides or N^ε^-retinyl-Lys, respectively, to capture any binding that was not detected by UV-Vis spectral measurements. However, LC-MS/MS analysis revealed no N^ε^-retinyl-peptides or N^ε^-retinyl-Lys, further supporting the lack of pigment formation with retinal in bRRH (Fig. 7*B*).

The same LC-MS/MS analyses were performed for bRRH kept in membrane isolates, unperturbed by the addition of LMNG detergent and depletion of membrane lipids. Membrane isolates of HEK293S GnTI^-^ with and without stable expression of bRRH were treated with different isomers of retinal. LC-MS/MS analyses displayed nonspecific binding with negligible levels of N^ε^-retinyl-peptides or N^ε^-retinyl-Lys near baseline levels from membrane isolates of either HEK293S cells with or without bRRH expression (Fig. 7, *C* and *D*). Thus far, no studies have conclusively demonstrated pigment formation with mammalian RRH. Based on our results, its role in the photoconversion of retinal in vertebrate systems remains unclear.

## Discussion

Hydrolysis of all-*trans* agonist from photoactivated Rho and cone opsins bridges two crucial cycles that are responsible for continuous vision: the classical visual cycle and the opsin photocycle. The chromophore release has been thoroughly studied with Rho but not nearly as rigorously explored in cone opsins, which are crucial for our daylight vision. Cone opsins are underexplored for several reasons, including the lack of adequate and available mammalian sources rich in cone opsins and difficulties with heterologous expression (26, 27). All-*trans* agonist hydrolysis from photoactivated cone opsin has largely been investigated indirectly using UV-Vis or fluorescence spectroscopy and electrophysiology (5, 6, 7). As shown by the current study, our LC-MS/MS-based method of direct monitoring of the retinylidene covalent linkage allowed the characterization of agonist hydrolysis in photoactivated cone opsins. Our findings that photoactivated cone opsins hydrolyze their agonist much more rapidly than photoactivated Rho aligns with previous spectral and electrophysiological findings. The much quicker release of all-*trans*-retinal indicates a faster photocycle or rates of hydrolysis and regeneration for cone opsins than for Rho. Indeed, in biochemical experiments, the decay of photoactivated cone pigment takes seconds, whereas minutes for Rho (28). In electrophysiological studies by Hecht, full cone dark adaptation occurs around 10 times faster than full rod dark adaptation, requiring over 30 min (11). Furthermore, rods saturate under dim light conditions, while cones can continue to be responsive under very bright light conditions, suggesting an alternative rapid supply of 11-*cis*-retinal to cones mediated by light (29, 30, 31). This alternative pathway of chromophore production by light was recently shown to be facilitated by RGR (15).

RGR in native RPE microsomal membranes has been shown to rapidly produce 11-*cis*-retinal from all-*trans*-retinal. Following the photoconversion of the all-*trans*-retinylidene adduct, the 11-*cis* product is quickly hydrolyzed, and all-*trans*-retinal is subsequently taken up promptly from the milieu. As such, under constant daylight illumination of the retina, RGR can plausibly serve to rapidly turnover all-*trans*-retinal to meet the demands for 11-*cis*-retinal by the cones. This role of RGR has recently been elucidated *in vivo* by electrophysiological studies investigating cone function with a mouse model designed to conditionally activate RGR expression (15). These observations overall indicate RGR to be a monostable nonvisual opsin with *trans-cis* photoisomerization activity. Recent studies have suggested the bistability of RGR (32), which is a plausible interpretation under the conditions used (RGR in detergent micelles). Considering our findings that the hydrolysis of the 11-*cis*-retinylidene adduct is much slower in RGR immunopurified in detergent micelles as compared to native microsomal membranes, the delayed hydrolysis rate in detergent would allow time for another photoisomerization event, thereby conferring an apparent bistable nature.

The effect of detergent micelles to delay hydrolysis of the photoisomerized retinylidene products in opsin proteins was previously observed for photoactivated Rho (9). The hydrolysis of Rho and RGR photoproducts in detergent as compared to native membranes was slowed to around half (9) and to under a sixth, respectively. Given that detergent micelles are commonly used to study membrane proteins, our results highlight an important observation about the artifactual effects detergents have on membrane protein biochemistry. These effects are further emphasized by our findings that detergents decreased the thermostability of both Rho and RGR as compared to native membranes.

Recently, a nanobody that targets the N terminus and extracellular loop 2 of bRho was shown to significantly decrease the rate of hydrolysis of the all-*trans*-agonist in photoactivated bRho (33). In a panel of monoclonal anti-bRGR antibodies developed in-house, several were identified to successfully target bRGR, specifically at the N and C termini. Similarly to the anti-bRho nanobody, the anti-N-terminal bRGR antibodies delayed hydrolysis, while the anti-C-terminal bRGR antibodies had a negligible effect on hydrolysis. This observation corroborates the N terminus as a key region that appears to modulate the Schiff-base hydrolysis of opsin photoproducts, whether bRho or bRGR.

Like RGR, RRH resides in the RPE but in the apical membrane surface of the RPE as opposed to microsomes. This location would be ideal for the capture of all-*trans*-retinal from photoreceptor outer segments, if it exhibited pigment formation and *trans*-*cis* photoisomerization activity similar to RGR. However, such activity by RRH has only been confirmed for invertebrate homologs (17, 18). Instead, our study showed that bRRH, unlike bRGR, was unable to form pigment with all-*trans*-retinal or any of the three major mono-*cis* isomers of retinal (9-*cis*, 11-*cis*, or 13-*cis*), despite possessing an internal Lys residue within the putative chromophore-binding domain. Our result is consistent with previous studies that have shown the lack of a robust phenotype in RRH knockout mice (34). Furthermore, our analysis of single-cell RNA sequencing data showed very low expression levels of RRH, which was corroborated by proteomics data showing very low RRH protein levels, relative to other proteins from the RPE. One study reported RRH expression in ocular tissue during early development of human and mouse eyes, with expression prior to Rho, cone opsins, and RGR (25). However, given the lack of a significant phenotype in RRH knockout mice, the exact role of RRH either in development or in the visual cycle remains unclear. Another study has suggested a potential role for RRH in trafficking retinoids to the RPE from photoreceptors in mice, perhaps even guiding visual cycle directionality (34). However, the lack of expression within other vertebrates, like humans and cows, to the extent found in mice and significantly lower relative expression as compared to other retinoid carrier proteins, such as cellular retinaldehydes-binding protein 1 and interphotoreceptor retinoid-binding protein, could suggest an alternative role to retinoid trafficking. Furthermore, RRH also lacks the NPXXY motif implicated for G-protein signaling.

**Figure 8:**
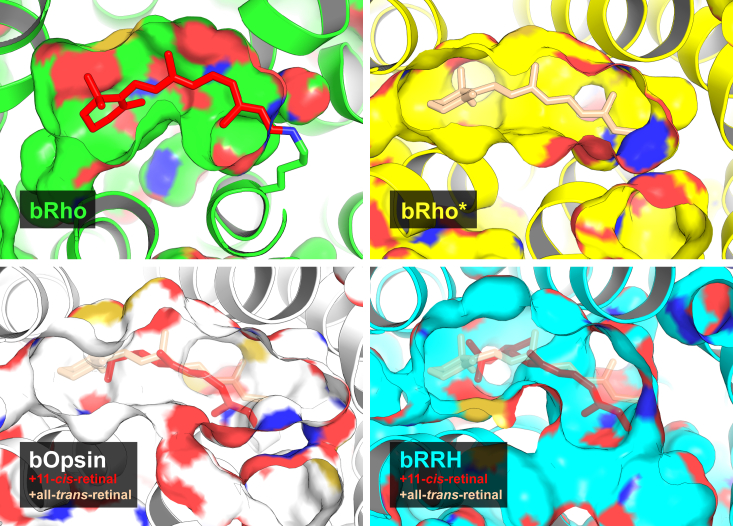
**Structural analysis of the bRRH putative chromophore-binding pocket.** Comparison of the ligand-binding pockets in ground-state rhodopsin (bRho, *top left*), metarhodopsin II (bRho∗, *top right*), apo-rhodopsin (bOpsin, *bottom left*), and peropsin (bRRH, *bottom right*). The crystal structures of bRho∗, bOpsin, and the AlphaFold model of bRRH were superimposed to the crystal structure of bRho and depicted in surface representation. The models of bOpsin and bRRH include 11-*cis*-retinal from bRho and all-*trans*-retinal from bRho∗ (semitransparent sticks), for reference.

For a better understanding of bRRH, a comparative structural study was performed by overlaying the predicted AlphaFold structure of bRRH with the crystal structures of bRho, its photoactivated state, and apo-opsin (Fig. 8). From the structural alignment with bRho and its photoactivated state, bRRH appears to have a binding pocket with sufficient space to fit either 11-*cis*-retinal or all-*trans*-retinal. Given that the 3D model was calculated using bRRH devoid of ligand, the same structural alignments were performed for apo-opsin, which resulted in a better fitting for all-*trans*-retinal than for 11-*cis*-retinal (Fig. 8).

The observation that bRRH has ample space to bind retinoids, an internal Lys residue (K284) within the putative chromophore-binding domain and an available counterion (D169) for a protonated Schiff base contrasts with our results showing lack of pigment formation with any of retinal isomers. One possible explanation would involve the presence of bulk water that could participate in the destabilization and hydrolysis of any retinylidene Schiff base that could form within bRRH, similarly to the role of bulk water in photoactivated Rho (35, 36). To explore this possibility, the binding pocket of bRRH was compared to that of invertebrate (*Hasarius adansoni*) RRH, known to form stable pigment with bistable retinylidene photochemistry. The comparison demonstrated that a wider binding pocket of bRRH that is more accessible to bulk water as compared to invertebrate RRH (Fig. S18).

Overall, given the findings from the comparative structural study and our LC-MS/MS analysis of proteolyzed bRRH treated with various isomers of retinal, the role of RRH in retinylidene photoisomerization in vertebrate systems remains largely unclear. These negative results of RRH contrast with the positive identification of Schiff base formation in the case of bRGR and the three cone opsins. Since the experiments for the four heterologously expressed opsins were done in similar conditions, we can safely rule out the existence of a systematic artifact of our methodology for the case of bRRH.

In summary, our study provides direct measurements of hydrolysis of agonist from photoactivated cone opsins, confirming the quick photocycle of cone opsins and documenting the large demand for 11-*cis*-retinal and the hefty production of all-*trans*-retinal to be cleared and recycled by RGR and/or the classical visual cycle providing a consistent supply of chromophore under constant illumination to support to sustain photopic vision. Upon probing RGR biochemistry, the N terminus and the lipid environment surrounding the transmembrane helices appeared to strongly influence hydrolytic production of 11-*cis*-retinal following *trans-cis* photoisomerization. Our study also highlights the importance of studying membrane proteins in their native membrane environments, especially when investigating biochemical rates or activity. Lastly, our study demonstrated the lack of RRH pigment formation for subsequent photoisomerization activity, as well as highly variable expression in the mammalian RPE, leaving an unanswered question on the role of RRH in the eye.

## Experimental procedures

### Proteinase K and pronase digestion of opsin pigments

Opsin pigments were prepared in a dark room under dim red light, at 1 to 2 mg/ml in 10 mM 4-(2-hydroxyethyl)-1-piperazine ethanesulfonic acid (Hepes), pH 7.4, containing 140 mM NaCl. One volume of opsin pigment solution was treated with two to three volumes of saturated NaBH_4_ or NaBD_4_ in ice-cold *i*PrOH for immediate reduction of the retinylidene Schiff base and isolation of reduced opsin pigment by *i*PrOH protein precipitation (cold ethanol [EtOH] can be used in place of *i*PrOH). The suspension of protein precipitate was diluted with three parts cold methanol (MeOH) and 1 part 50 mM Hepes buffer, pH 7.4, to facilitate quenching of unreacted NaBH_4_ or NaBD_4_. After centrifugation at 20,000*g*, the protein pellet was washed again with cold MeOH, followed by cold water. The protein pellet was resuspended in either proteinase K buffer (100 mM bis-tris propane, pH 7.8, 100 mM CaCl_2_, 4 M urea) or pronase buffer (100 mM bis-tris propane, pH 7.8, containing 100 mM CaCl_2_, 0.5% w/v CHAPS). Then, proteinase K or pronase were added at approximately 10 times the weight of the opsin substrate. The proteinase K digestion mixture was incubated at room temperature while the pronase digestion mixture was incubated at 6 to 10 °C. Both digestions proceeded for 24 h with gentle agitation using a shaker. Each digest was passed through a BioPureSPN C18 spin column for desalting. The column was washed with 20% acetonitrile (ACN) in water with 0.1% formic acid (FA), and peptides were eluted using 50% ACN. The eluted N^ε^-retinyl-peptide or N^ε^-retinyl-Lys products were separated using a Dionex UHPLC with a XBridge C18 column and a 40 min gradient of 20% to 60% ACN in water with 0.1% FA at a flow rate of 0.3 ml/min. The N^ε^-retinyl products were detected by high-performance liquid chromatography (HPLC) absorbance measurements at 330 nm and identified by LC-MS/MS with collision-induced dissociation fragmentation, using an LTQ XL mass spectrometer.

### Kinetic analysis of Schiff-base hydrolysis of opsin pigment photoproducts

Each opsin pigment in 10 mM Hepes, pH 7.4, containing 140 mM NaCl was illuminated for 10 s with a fiber-coupled monochromatic LED at an intensity of 125 μW at wavelengths indicated as follows: 505 nm for Rho, 565 nm for red opsin, 530 nm for green opsin, 455 nm for blue opsin, and 530 nm for bRGR. After light exposure, NaBH_4_/*i*PrOH was added at different timepoints to track the hydrolysis of the photoisomerized retinylidene Schiff base of each opsin protein. The protein precipitate was digested with pronase as described above, producing N^ε^-retinyl-Lys to determine the amount of remaining opsin-bound retinal. The supernatant was analyzed to quantify the hydrolytically released retinal as follows: all-*trans*-retinol, the NaBH_4_-reduction product of all-*trans*-retinal, was separated and quantified by HPLC using the Agilent 1260 Infinity HPLC system with an XBridge C18 column with a gradient in water with 0.1% FA of 95% to 100% of MeOH with 0.1% FA for 10 min at a flow rate of 0.3 ml/min. In the study of cone opsins, the Schiff-base hydrolysis of the photoproducts was studied at different temperatures (0 °C and 20 °C), given the rapid rate of hydrolysis during the 10 s illumination period. The resultant extents of hydrolysis were compared to those of Rho under the same conditions. In the study of bRGR, a different HPLC method was used to separate the 11-*cis*-retinol product of NaBH_4_-reduction from all-*trans*-retinol, because all-*trans*-retinal had been added in slight excess to initially regenerate the bRGR pigment. Using the same HPLC system and column, the procedure entailed a gradient in water with 0.1% FA of 80% to 100% MeOH with 0.1% FA for 15 min at a flow rate of 0.3 ml/min. From the pronase digest, the resultant N^ε^-retinyl-Lys products were analyzed chromatographically using the same HPLC system and column, but a faster method was used with an 18-min gradient of 30% to 39% ACN in water with 0.1% FA at a flow rate of 0.3 ml/min. The detection of N^ε^-retinyl-Lys products by HPLC absorbance detection at 330 nm and LC-MS/MS were done as described above. Isomeric identities were confirmed by UV-Vis absorbance spectra collected during chromatography. The molar quantity of each isomer of N^ε^-retinyl-Lys (9-*cis*, 11-*cis*, 13-*cis*, and *all-trans*) was determined based on a standard curve generated using synthetically produced N^ε^-retinyl-Lys isomers, as described previously (9). The mole fraction of opsin-bound retinal (measured as moles of N^ε^-retinyl-Lys) over the total retinal content (measured as the sum of the moles of N^ε^-retinyl-Lys and the moles of retinal produced from hydrolysis of retinylidene Schiff base) was plotted for each time point, generating a curve consistent with pseudo first-order decay kinetics.

### Expression and purification of opsin proteins

Details about the expression and purification of mammalian cone opsins, bRRH, and bRGR are provided in the Supplemental Methods. Isolation of bRPE microsomes, proteomics of bRPE cells, and generation of bRGR antibodies are also described in the Supplemental Methods.

## Data availability

All data from this study are included in the main article and/or the Supplement.

## Supporting information

This article contains supporting information (37, 38, 39, 40, 41, 42, 43, 44, 45, 46, 47, 48, 49, 50).

## Conflict of interest

K. P. is a consultant for Polgenix Inc. and AbbVie Inc. and serves on the Scientific Advisory Board of Hyperion Eye Ltd. The other authors declare that they have no conflicts of interest with the contents of this article.
