# Supplementary material for: Retinylidene chromophore hydrolysis from mammalian visual and non-visual opsins
Source: J Biol Chem. 2024 Jan 23;300(3):105678. doi: 10.1016/j.jbc.2024.105678 (PMC10877631; doi:10.1016/j.jbc.2024.105678)
Supplement: Supporting information [file mmc1.pdf]

SUPPLEMENT

Retinylidene Chromophore Hydrolysis from Mammalian Visual and Non-Visual Opsins

John D. Hong [1, 2], David Salom [1], Elliot H. Choi [1], Samuel W. Du [1, 3], Aleksander Tworak [1], Roman Smidak [1], Fangyuan Gao [1], Yasmeen J. Solano [1, 3], Jianye Zhang [1], Philip D. Kiser [1,3,4,5], Krzysztof Palczewski [1,2,3,6]

1. Gavin Herbert Eye Institute, Department of Ophthalmology, University of California Irvine, Irvine, CA 92697, USA
2. Department of Chemistry, University of California Irvine, Irvine, CA 92697, USA
3. Department of Physiology and Biophysics, University of California Irvine, Irvine, CA 92697, USA
4. Department of Clinical Pharmacy Practice, University of California Irvine, Irvine, CA 92697, USA
5. Research Service, VA Long Beach Healthcare System, Long Beach CA, 90822, USA
6. Department of Molecular Biology and Biochemistry, University of California Irvine, Irvine, CA 92697, USA

Corresponding Author:

Krzysztof Palczewski, Department of Ophthalmology, Gavin Herbert Eye Institute, UCI, Irvine, CA 92697; kpalczew@uci.edu; phone (949) 824-6527

Table of Content

1. Instrumentation
2. Materials
3. Supplementary methods
4. Supplementary figures 1-17

Instrumentation

HPLC System (Agilent 1100 Series, Santa Clara, CA); 1260 Infinity HPLC System (Agilent); Dionex Ultimate 3000 UHPLC System (Thermo Fisher Scientific, Waltham, MA); LTQ XL mass spectrometer (Thermo Fisher Scientific); Cary 50 UV-Vis Spectrophotometer (Varian, Palo Alto, CA); Isotemp 3016S Water Bath (Fisher Scientific, cat no. 13-874-28); Fiber-coupled 565-nm 9.9-mW (Min) 700-mA LED (Thorlabs, Newton, NJ, cat. no. M565F3); Fiber-coupled 530-nm 6.8-mW (Min) 1000-mA LED (Thorlabs, cat. no. M530F2); Fiber-coupled 455-nm 17-mW (Min) 1000-mA LED (Thorlabs, cat.no. M455F3); Fiber Patch Cable (Thorlabs, cat. no. M92L01); Compact T-Cube 1200-mA LED Driver with Trigger Mode (Thorlabs, cat. no. LEDD1B); Power Supply Unit with 3.5-mm Jack Connector (Thorlabs, cat. no. KPS201).

Materials

Chemicals and other materials were purchased as follows: all-*trans*-retinal (Sigma-Aldrich, St. Louis, MO, cat. no. R2500), all-*trans*-retinol (Sigma-Aldrich, cat. no. R7632); bROS (InVision Bioresources, Seattle, WA, cat. no. 98740); 2-[4-(2-hydroxyethyl)piperazin-1-yl]ethanesulfonic acid (Goldbio, St. Louis, MO, cat. no. H-400-1); 1,3-bis[tris(hydroxymethyl)methylamino]propane (Sigma-Aldrich, cat. no. B6755); calcium chloride dihydrate (Sigma-Aldrich, cat. no. 223506); n-dodecyl-β-D-maltopyranoside sol-grade (Anatrace, Maumee, OH, cat. no. D310S); lauryl maltose neopentyl glycol (Anatrace, Maumee, OH, cat. No. NG310); (3-((3-cholamidopropyl) dimethylammonio)-1-propanesulfonate) CHAPS detergent Anagrade (Anatrace, OH, cat. no. C316); pronase (Sigma-Aldrich, cat. no. PRON-RO); proteinase K solution (Viagen Biotech, cat. no. 501-PK); Amicon Ultra-0.5 Centrifugal Filter (Millipore, Burlington, MA, cat. no. UFC503096); ACN Optima LC/MS grade (Fisher Scientific, Pittsburgh, PA, cat. no. A955); methanol Optima LC/MS grade (Fisher Scientific, cat. no. A456); isopropanol Optima LC/MS grade (Fisher Scientific, cat. no. A461); FA Optima LC/MS grade (Fisher Scientific, cat. no. A117-50); methylene chloride (HPLC grade) (Fisher Scientific, cat. no. D143); hexanes (HPLC grade) (Fisher Scientific, cat. no. H302); ethyl acetate (HPLC) (Fisher Scientific, cat. no. E195); triethylamine (HPLC) (Fisher Scientific, cat. no. O4884); dimethyl sulfoxide (Sigma-Aldrich, cat. no. D2650); N,N-dimethylformamide (Fisher Scientific, cat. no. AC327171000); diethylamine (Sigma-Aldrich, cat. no. 471216); N^{α}-[(9H-fluoren-9-ylmethoxy)carbonyl]-L-Lys hydrochloride (Fisher Scientific, cat. no. F0586); 1,2-dioleoyl-sn-glycero-3-phosphoethanolamine (Avanti Polar Lipids, Alabaster, Al, cat. no. 850725P); $NaBH_4$ (Alfa Aesar, Haverhill, MA, cat. no. 88983); $NaBD_4$ (Alfa Aesar, cat. no. 35102; 85 W 2700 K Hg light bulb (Home Depot, cat. no. EDXR-40-19); BioPureSPN C18 TARGA (nestgrp, Ipswich, MA cat. no. HEM S18R); XBridge C18

(Waters, Milford, MA, 2.1 mm X 100 mm, cat. no. 186003022); Gemini Analytical C18 (Phenomenex, Torrance, CA, 250 x 4.6 mm, cat. no. 00G-4435-E0); Gemini Preparatory C18 (Phenomenex, 250 x 10 mm, cat. no. 00G-4435-N0); Luna Preparatory Silica (Phenomenex, 250 x 21.2 mm, cat. no. 00G-4091-P0-AX).

Supplementary methods

Production of 9-*cis*-, 11-*cis*-, and 13-*cis*-retinal and the corresponding retinol isomers. The method for production of 9-*cis*-retinal, 11-*cis*-retinal, and 13-*cis*-retinal was adapted from Kahremany *et al.* (37). Thus, all-*trans*-retinal was solubilized in ACN at 0.2 M and illuminated with an 85-W mercury light bulb at maximum intensity at a distance of 10 cm for 12 hr at 4 °C. The resultant 9-*cis*-, 11-*cis*-, and 13-*cis*-retinal products were purified with a Luna preparatory silica column using 90% hexane and 10% ethyl acetate as the mobile phase at a flow rate of 5 mL/min. The purified products were verified by comparison to standards, using HPLC and UV-Vis absorbance spectroscopy. Each retinal isomer was reduced with excess $NaBH_4$ in methanol to produce the corresponding retinol isomer. The methanol solvent was evaporated by a flow of nitrogen, and the corresponding retinol product was isolated from the crude oil by organic-aqueous extraction using 1:1 hexane/water. The hexane layer was dried with anhydrous magnesium sulfate. The crude product was purified with a Luna preparatory silica column, using 90% hexane and 10% ethyl acetate as the mobile phase at a flow rate of 5 mL/min. The purified products were verified by comparison to standards, using HPLC and UV-Vis absorbance spectroscopy.

Isolation of bROS membranes. bROS membranes were prepared from fresh bovine retinas (InVision Bioresources) using sucrose gradient isolation as described previously (38), and washed with hypotonic buffer (20 mM HEPES, pH 7.4).

Expression of red-cone opsin. The red-cone opsin (OPSR) construct consisted of human long-wavelength-sensitive opsin 1 (UNIPROT ID P04000) in which the C-terminus was truncated after Lys352 and tagged with 1D4 peptide, and residues Gln253-Lys254 from the third intracellular loop were replaced by a modified T4 lysozyme (UNIPROT ID P00720, with M1 and its three C-terminal residues truncated, and mutations C54T and C97A). The OPSR construct was expressed in-house in Sf9 insect cells as described previously (26,39). The OPSR construct was cloned into the pFastBac HT vector from a Bac-to-Bac Vector Kit (Thermo Scientific, Waltham, MA) with their N-terminal His tags removed. DH10Bac *E. coli* was transformed using the resultant recombinant plasmid to produce bacmid, and isolated by mini-

prep following the Invitrogen protocol for the Bac-to-Bac Baculovirus Expression System (Carlsbad, CA). Sf9 insect cells were transfected during log-phase growth with 1 μg of bacmid DNA. The supernatant was harvested after 5 days. 1.5 mL of P1 virus was added to 40 mL of cells at a density of 1.5 × 10^6 cells/mL. After 4 days, the culture was centrifuged at 2,500 x g for 5 min to harvest the P2 virus. P2 virus (2 mL) was added to 800 mL of Sf9 cells at a density of 2 × 10^6 cells/mL to obtain P3 virus. Large-scale expression of OPSR was started with Sf9 cells at 3.5 × 10^6 cells/mL of culture. Sf9 cells were subsequently infected with P3-stage virus at a 1:100 volume ratio in 3-liter flasks with shaking at 135 rpm in a 27.5 °C incubator for 2 days. Then cells were collected 48 hr post-infection. Cell pellets were frozen and stored at -80 °C.

Expression of green-cone opsin. The green-cone opsin (OPSG) construct consisted of the human medium-wavelength-sensitive opsin 1 (UNIPROT ID P04001) in which the C-terminus was truncated after residue Lys-352, and 1D4-tagged. Expression of OPSG in insect Tni cells was conducted by Expression Systems LLC (Davis, California, US). Cell cultures were seeded at 1 x 10^6 cells/mL in ESF 921 medium, and cultured overnight. The following day the culture was infected with baculovirus at a MOI of 10:1. The culture was harvested 72 hr post infection. Cells were pelleted by centrifugation and frozen at -80 °C.

Expression blue-cone opsin. The blue-cone opsin (OPSB) construct consisted of human short-wavelength-sensitive opsin 1 (UNIPROT ID P03999) in which the C-terminus was truncated at residue Lys^{334}, and replaced with the 1D4 tag. The OPSB construct was expressed in-house in Sf9 insect cells, as described above for OPSR.

Generation of HEK293S $GnTI^-$ cell line stably expressing bRGR. A HEK293S $GnTI^-$ cell line stably expressing bRGR (UNIPROT ID P47803) was established using a previously published method (40). Retroviral expression vectors were generated by introducing EcoRI and NotI restriction sites at the ends of the bRGR coding sequence by PCR with the following primers: forward, 5'-CGGGAATTCATGGCAGAGTCTGG-3; reverse: 5'-CGAATGCGGCCGCTTAGGCAGGCGCCACTTGG-3'. Subsequently, the bRGR cDNA was inserted into a pMX-IP retroviral vector, generously provided by Dr. T. Kitamura from the University of Tokyo (41).

An internal ribosomal entry site (IRES) and a puromycin selection gene were placed downstream of the bRGR coding region, enabling puromycin-based selection. HEK293S $GnTI^-$ cells were transduced with retrovirus, which was harvested from Phoenix-AMPHO cells transfected with the pMXs-IP vector carrying the bRGR sequence. Following three days of

selection with puromycin, the transduced cells were maintained with FreeStyle 293 expression medium (Thermo Fisher Scientific; 12338018) containing 3% v/v heat-inactivated fetal bovine serum (Thermo Fisher Scientific; A3840001), 1% v/v Penicillin/Streptomycin mix (Thermo Fisher; 151401122). Suspension cells were maintained at 37 °C under an atmosphere of air adjusted to 8% CO_2.

Generation of HEK293S GnTI$^-$ cell line stably expressing bRRH. A HEK293S GnTI$^-$ cell line stably expressing bRRH (UNIPROT ID F1MR98) was established by transduction of GnTI-HEK293S cells with retrovirus obtained from Phoenix-Ampho cells, transfected with either pMXs-bRRH-WT-IRES-GFP, according to a previously published protocol (42). Transduced cells were sorted by a FACSAria Fusion cell sorter (BD Biosciences) to selectively collect transduced GFP+ cells. WT bRRH with a C-terminal 1D4 tags and flanking EcoRI and NotI restriction sites were synthesized and cloned into pcDNA3.1+ by GenScript. The vectors were then digested with EcoRI and NotI and cloned into the MCS of pMXs-IRES-GFP (a gift from T. Kitamura at the University of Tokyo (41)). The downstream sequence of the internal ribosomal entry site (IRES) and GFP allows co-expression of bRRH and GFP, thereby enabling cell sorting by flow cytometry. Cells were cloned by limited dilution and the expression of RRH was determined by 1D4 immunoblotting. Clones were then adapted to FreeStyle 293 expression medium (Thermo Fisher Scientific; 12338018) containing 3% v/v heat-inactivated fetal bovine serum (Thermo Fisher Scientific; A3840001), 1% v/v Penicillin/Streptomycin mix (Thermo Fisher; 151401122). Suspension cells were maintained at 37 °C under an atmosphere of air adjusted to 8% CO_2.

Immunopurification of retinylidene-opsin pigments. Frozen cell pellets were thawed and homogenized in hypotonic buffer (20 mM HEPES, pH 7.0, containing 1 mM $MgCl_2$, benzonase nuclease, Roche protease inhibitor cocktail) with a Dounce homogenizer. Membranes were isolated by centrifugation at 50,000 x g for 1 hr, then homogenized further in the same hypotonic buffer. The resultant membranes were then washed twice in hypertonic buffer (50 mM HEPES, pH 7.0, 1 M NaCl, 1 mM $MgCl_2$, benzonase nuclease, Roche protease inhibitor cocktail). The washed membranes were resuspended in hypertonic buffer, and incubated for 2 hr with retinal. 11-*cis*-retinal was used for cone opsins, and all-*trans*-retinal was used for bRGR. All four retinal isomers were tested for binding to bRRH, as a native chromophore has not been identified. All four retinal were prepared at 20 mM concentration in dimethylformamide for addition to membranes. After incubation, membranes were solubilized in a buffered-detergent solution (final concentration: 10 mM Lauryl Maltose Neopentyl Glycol (LMNG), 20 mM HEPES, pH 7.4,

0.25 M NaCl). For bRho, bROS membranes, isolated as described above, were solubilized similarly. Each retinylidene-opsin pigment was immunopurified in a dark room under dim red light, using immobilized 1D4 antibody as previously described (43-45). Solubilized membranes were incubated for 2 hr with Sepharose beads with immobilized 1D4 antibody. The beads were washed with 10 column volumes of wash buffer (0.2 mM LMNG in 20 mM HEPES, pH 7.4 containing 0.15 M NaCl) to remove unbound protein as well as solubilized lipids. The retinylidene-opsin pigment was then eluted using a 0.5 mg/mL solution of the competing peptide TETSQVAPA (1D4) in wash buffer. Using an Amicon 30-kDa MWCO centrifugal filter, retinylidene-opsin pigments were concentrated to at least 1 mg/mL while removing 1D4 peptide with two passes of wash buffer B (0.1 mM LMNG, 20 mM HEPES, pH 7.4, 0.14 M NaCl).

Isolation of bRPE microsomes. The preparation of bRPE microsomes was done as previously described (46). RPE cells were collected from bovine eye cups by gentle brushing, then lysed using a Dounce homogenizer. bRPE microsomes were isolated from the homogenate by differential centrifugation at 20,000 x *g* for 20 min at 4 °C, yielding a supernatant fraction containing microsomes that were pelleted by subsequent centrifugation at 150,000 x *g* for 1 hr at 4 °C. The microsomal pellet was resuspended in 10 mM HEPES buffer, pH 7.4, at a protein concentration of about 5 mg/mL containing about 0.2 mg/mL bRGR regenerated with all-*trans*-retinal.

Immunization protocol and hybridoma production for antibodies against bRGR. Purified bRGR was diluted to a concentration of 1.0 mg/mL in sterile PBS. Adult female C57/B6 mice (Jackson Laboratories) at 6-8 weeks of age, were injected intraperitoneally with 100 µl of PBS containing bRGR, mixed at a 1:1 (v/v) ratio with QuickAntibody Adjuvant (Beijing BIodragon Immunotechnologies). Boosters of the same volume were injected into the mice 1,4, and 5 weeks after the initial immunization. A week after the final injection, serum titers were evaluated by ELISA. Animals producing high-titer antibody were sacrificed for hybridoma production. For the development of hybridoma and monoclonal antibody production, the ClonalCell™-HY Hybridoma kit (Stem Cell Technologies) was used according to the manufacturer's instructions. Monoclonal antibodies derived from the selected hybridoma clones were evaluated using an immunoblot of lysates of HEK293S GnTI$^-$ bRGR and immunochemistry of HEK293S GnTI$^-$ bRGR.

Effect of anti-RGR antibodies on the hydrolysis of bRGR photoproduct. Each bRGR antibody was prepared at 0.5 mg/mL with 10 mM HEPES buffer, pH 7.4. One part by volume of bRPE microsomes were mixed with one part 2 % w/v LMNG, followed by two parts 0.5 mg/mL

bRGR antibody or buffer (10 mM HEPES buffer, pH 7.4). The mixture was incubated for 1 hr at room temperature. Aliquots of the mixture in a 20 °C water bath were illuminated by 10 sec of 125-uW 530-nm fiber light, then left to incubate in the dark for 1 min. The mixture was immediately placed on ice and treated with $NaBH_4$/*i*PrOH to determine extent of hydrolysis, as described above.

Thermostability studies of bRho and bRGR. All aliquots of bROS, bRPE microsomes, immunopurified bRho, and immunopurified bRGR were prepared in 20 mM HEPES, pH 7.4, containing 140 mM NaCl. Each were incubated for 10 min at various temperatures (20°C, 37°C, 50°C, 60°C, 70°C, 80°C, and 90°C), followed by addition of $NaBH_4$/*i*PrOH for chromophore trapping. The amount of chromophore bound to opsin at each temperature was determined by the signal intensity of Nε-retinyl-Lys after proteolysis by pronase as described above. The signal intensities at each temperature were normalized to that of 20°C, generating a sigmoidal plot of thermostability to determine the melting temperature for each opsin either in native membranes or in detergent micelles depleted of lipids.

Assessment of bRRH ability to form retinylidene-opsin pigment. The immunopurification of bRRH was performed as described above, testing each isomer of retinal (9-*cis*, 11-*cis*, 13-*cis*, and all-*trans*) and vehicle control (dimethylformamide). The immunopurified bRRH was prepared in 20 mM HEPES, pH 7.4, containing 140 mM NaCl. $NaBH_4$/*i*PrOH was added to trap any bound chromophore for subsequent LC-MS/MS analysis of any N^{ε}-retinyl-peptides or N^{ε}-retinyl-Lys by proteolysis with proteinase K or pronase, respectively, as described above.

Concurrently, HEK293S $GnTI^-$ with and without stable expression of bRRH was cultured in suspension and harvested. Both samples were homogenized in hypotonic buffer (20 mM HEPES, pH 7.0, containing 1 mM $MgCl_2$, benzonase nuclease, Roche protease inhibitor cocktail) with a Dounce homogenizer. Membranes were isolated by centrifugation at 50,000 x g for 1 hr, then homogenized further in the same hypotonic buffer. The resultant membranes were then washed twice in hypertonic buffer (50 mM HEPES, pH 7.0, 1 M NaCl, 1 mM $MgCl_2$, benzonase nuclease, Roche protease inhibitor cocktail).

Membranes from HEK293S $GnTI^-$ with and without stable expression of bRRH were each washed three times in 20 mM HEPES, pH 7.4, containing 140 mM NaCl, for final preparation of membranes in this buffered condition at a concentration of 1.5 mg/mL total protein. Membranes from the HEK293S stably expressing bRRH was determined to have 3.5 ug bRRH per mg of protein. The membranes from each cell line were treated with each isomer of retinal (9-*cis*, 11-

cis, 13-*cis*, and all-*trans*) or vehicle control (dimethylformamide). Retinal was incubated for at least 2 h, followed by addition of $NaBH_4$/*i*PrOH for LC-MS/MS analysis of any N^{ε}-retinyl-peptides or N^{ε}-retinyl-Lys by proteolysis with proteinase K or pronase, respectively, as described above.

Structural comparative analysis of the putative chromophore binding pocket of bRRH. In order to compare the hypothetical retinal binding pocket of peropsin to that of rhodopsin, the AlphaFold (47,48) 3D model of bRRH (UNIPROT ID F1MR98) and the crystal structures of metarhodopsin II (PDB ID 3PXO) and apo-rhodopsin (PDB ID 3CAP) were superimposed in Pymol Molecular Graphic System (v1.2, Schrödinger) to the crystal structure of ground state rhodopsin (PDB ID 7ZBC). In addition, the binding pockets of AlphaFold models for bRRH and jumping spider's (*Hasarius adansoni*) RRH (HaRRH, UNIPROT ID E1CFG1) were compared side-by-side on Pymol.

Proteomic analysis of bRPE cells. Bovine RPE cells were resuspended in different proteolysis buffers to assess whether buffer conditions alter proteolytic selectivity and resultant distribution of peptides. The three buffers tested were: UA buffer (8.0 M Urea, 0.1 M Tris-HCl, pH 8.3), SDS buffer 1 (0.1 % SDS, 0.1 M Tris-HCl pH 8.3), and SDS buffer 2 (4.0 % SDS, 0.1 M Tris-HCl, pH 8.3). Protease inhibitor cocktail (Bimake #14001) was added to each buffer before use. The respective suspensions were sonicated on ice for 4 min, followed by centrifugation at 12,000 x *g* for 10 min at 4 °C. The supernatants were collected and digested by the filter-aided sample preparation (FASP) method. Briefly, the supernatant was transferred into a spin filter column (30-kDa cutoff). Proteins were reduced with 10 mM DTT for 1 hr at 56 °C, and alkylated with 20 mM iodoacetic acid for 30 min at room temperature in the dark. Next, the buffer was diluted and exchanged with 50 mM NH_4HCO_3 by washing the membrane three times. Free trypsin was added into the protein solution at a trypsin to protein ratio of 1:50 and incubated for 4 hr at 37 °C. Next, the digestion mixture was mixed at a 1:1 ratio (v:v) with buffer containing 20 mM $CaCl_2$ and 200 mM Tris-HCl, pH 8.3). Free chymotrypsin was then added to the digestion mixture at a chymotrypsin to protein ratio of 1:50 and incubated overnight at room temperature. After centrifugation at 12,000 x *g*, the proteolysis products were recovered in the supernatant. The supernatant was vacuum-dried and then adjusted to 200 µL with 0.5% acetic acid. The peptide mixtures were then subjected to C18 solid-phase extraction (The Nest Group, Inc.) for desalting.

Mass spectrometric data acquisition. Proteomics data were acquired *via* LC-MS/MS using an UltiMate 3000 UHPLC (Thermo Fisher Scientific), coupled in-line with an Orbitrap Fusion Lumos mass spectrometer (Thermo Fisher Scientific) with an ESI nanospray source. Mobile phase A was composed of 0.1% FA in water, and mobile phase B was comprised of 0.1% FA in ACN.

The total flow rate was 300 nL min^{-1}, and peptides were separated over an 89-min gradient from 1% to 22% buffer B (total run time 120 min per sample) on an Acclaim PepMap RSLC column (50cm x 75 µm). Survey (MS) scans were acquired in Orbitrap (FT) with automated gain control (AGC) target 8E5, maximum injection time 50 msec, and dynamic exclusion of 30 sec across the scan range of 375-1800 m/z. MS/MS spectra were acquired in data-dependent acquisition mode with an inclusion list of predicted m/z values for the peptides of bRRH, at top speed for 3 sec per cycle (**Table 1**); the AGC target was set to 4E5 with maximum injection time of 35 msec. Ions were subjected to stepped-energy higher-energy collision dissociation (seHCD) fragmentation at a normalized collision energy (NCE) of 30%.

Table 1. Mass (m) and *to* charge (z) ratio for analyzed peptides.

**Peptide Sequence**	**m/z**
NNLGNSSDC(carbamidomethyl)K	554.738
SQTEHNIVAAY	616.799
LITAGVISILSNIIVLGIF	978.611
TPTNAIIINLAVTDIGVSSIGYPMSAASDLHGSW	1736.377
GMASIGLLTVVAVDR	751.424
LTIC(carbamidomethyl)HPDAGR	570.285
ISMILGAW	445.744
ALMPIIGW	450.754
APDPTGATC(carbamidomethyl)TINW	702.317
TMMVVAINF	513.262
IVPLTVMF	460.270
HVTQSIK	406.735
HHGTNNC(carbamidomethyl)TEY	616.7415
SDQVDVTK	446.225
MSVIMILMF	542.784
IPPSMAIIAPLF	635.367
C(carbamidomethyl)QTTQAMPVTSVLPMDVPQNPLTSGK	1400.688

Label-free quantification analysis. The raw LC-MS/MS data files were analyzed using MaxQuant (version 1.5.2.8), with the spectra searched against the Uniprot mouse database (updated on May 21st, 2018). For identification of the peptides, the mass tolerances were 20

ppm for initial precursor ions, and 0.5 Da for fragment ions. Two missed cleavages in tryptic digests were allowed. Cysteine residues were set as static modifications. Oxidation of methionine was set as the variable modification. Filtering for the peptide identification was set at a 1% false discovery rate (FDR).

Supplementary Figures

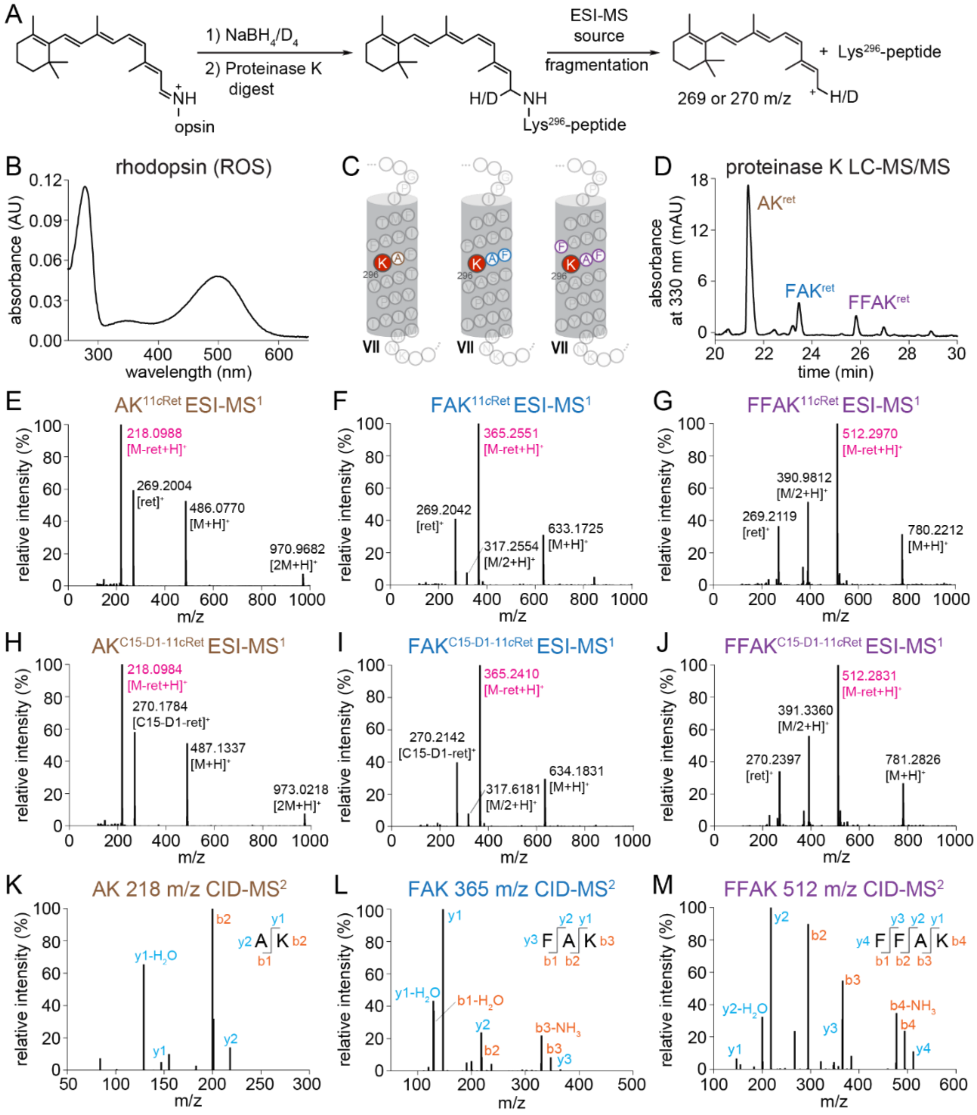

Figure S1. LC-MS/MS analysis of proteinase K digests of bRho from bROS membranes. (A) Schematic diagram of sample preparation and MS workflow. (B) UV-Vis spectrum of bRho in bROS solubilized in LMNG. (C) Location of chromophore-binding residue within helix VII of bRho, with labeled N$^{\varepsilon}$-retinyl-peptide fragments detected from the proteinase K digest. (D) Chromatographic separation of N$^{\varepsilon}$-retinyl-peptides from proteinase K digestion of bRho from bROS membranes, treated with $NaBH_4$ or $NaBD_4$ in *i*PrOH. (E-J) ESI-MS^1 spectra showing the characteristic cleavage of the retinyl cation from precursor retinyl-peptide analyte, producing a product peptide peak. (K-M) MS^2 spectra of CID fragmentation of product peptide ion for sequence determination.

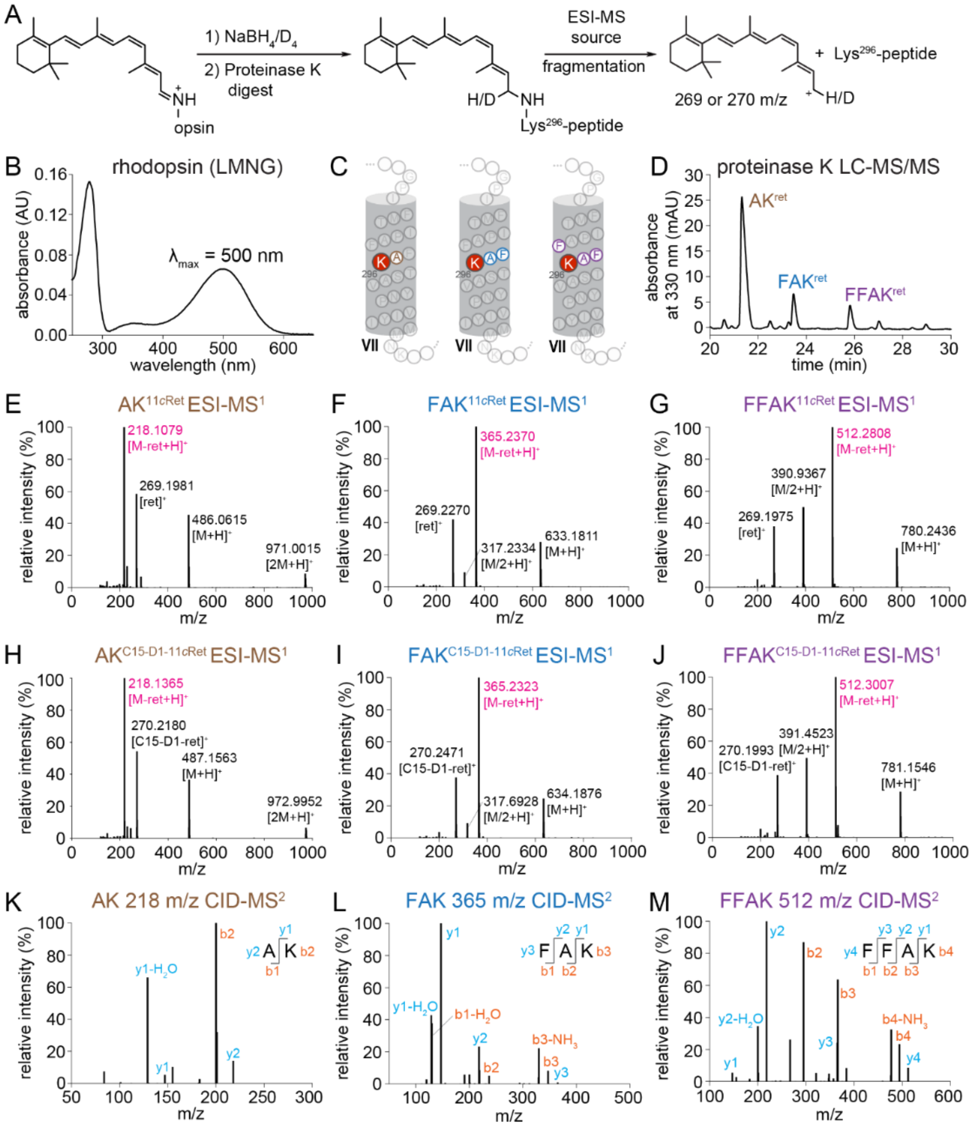

Figure S2. LC-MS/MS analysis of proteinase K digests of bRho purified in LMNG detergent micelles. (A) Schematic diagram of sample preparation and MS workflow. (B) UV-Vis spectrum of purified bRho in LMNG. (C) Location of chromophore-binding residue within helix VII of bRho, with labeled N^{ε}-retinyl-peptide fragments detected from the proteinase K digest. (D) Chromatographic separation of N^{ε}-retinyl-peptides from proteinase K digestion of purified bRho, treated with $NaBH_4$ or $NaBD_4$ in *i*PrOH. (E-J) ESI-MS^1 spectra showing the characteristic cleavage of the retinyl cation from precursor retinyl-peptide analyte, producing a product peptide peak. (K-M) MS^2 spectra of CID fragmentation of product peptide ion for sequence determination.

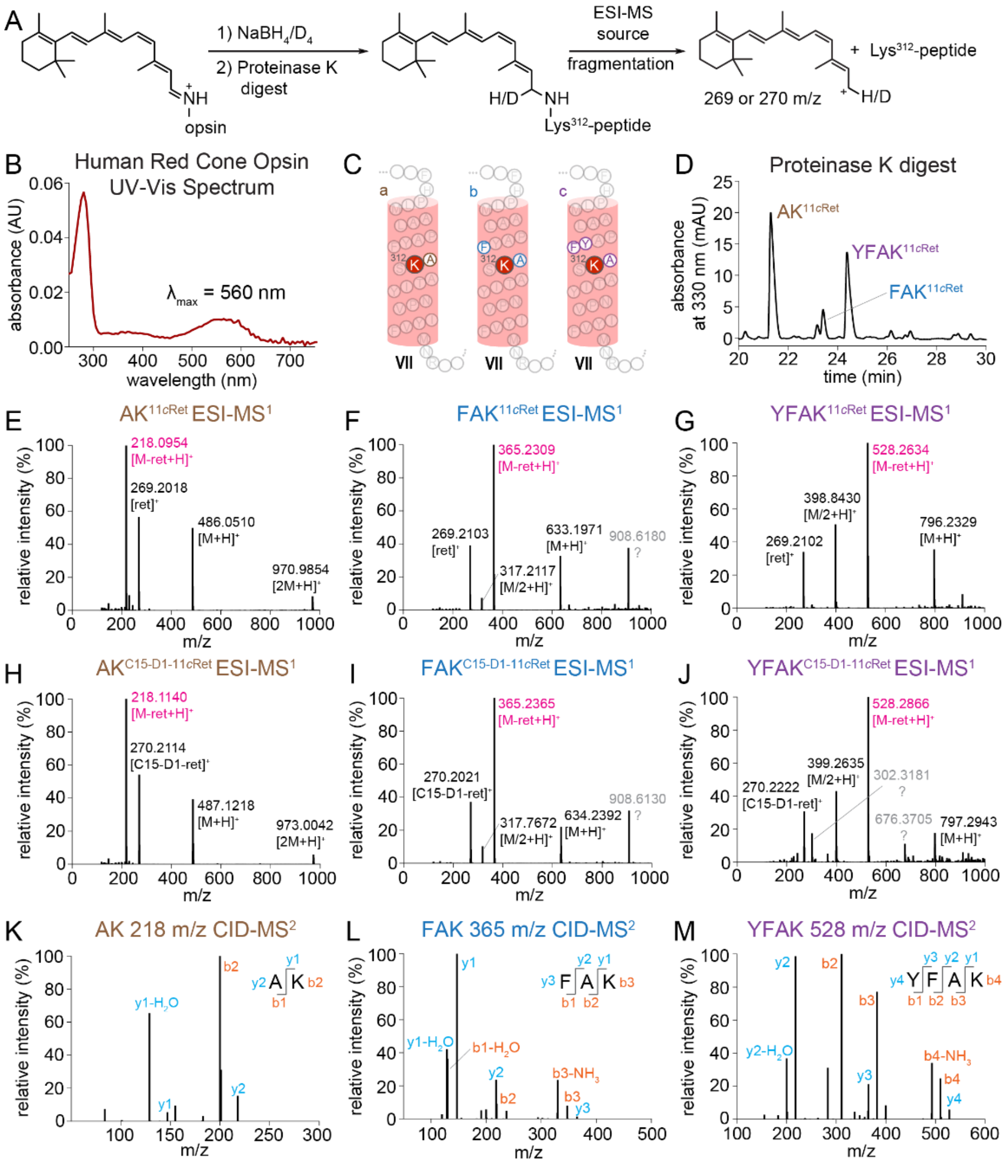

Figure S3. LC-MS/MS analysis of proteinase K digests of red cone opsin purified in LMNG detergent micelles. (A) Schematic diagram of sample preparation and MS workflow. (B) UV-Vis spectrum of purified red-cone opsin in LMNG. (C) Location of chromophore-binding residue within helix VII of red-cone opsin, with labeled N^{ε}-retinyl-peptide fragments detected from the proteinase K digest. (D) Chromatographic separation of N^{ε}-retinyl-peptides from proteinase K digestion of purified red-cone opsin, treated with $NaBH_4$ or $NaBD_4$ in *i*PrOH. (E-J) ESI-MS^1 spectra showing characteristic cleavage of the retinyl cation from precursor retinyl-peptide analyte, producing a product peptide peak. (K-M) MS^2 spectra of CID fragmentation of product peptide ion for sequence determination.

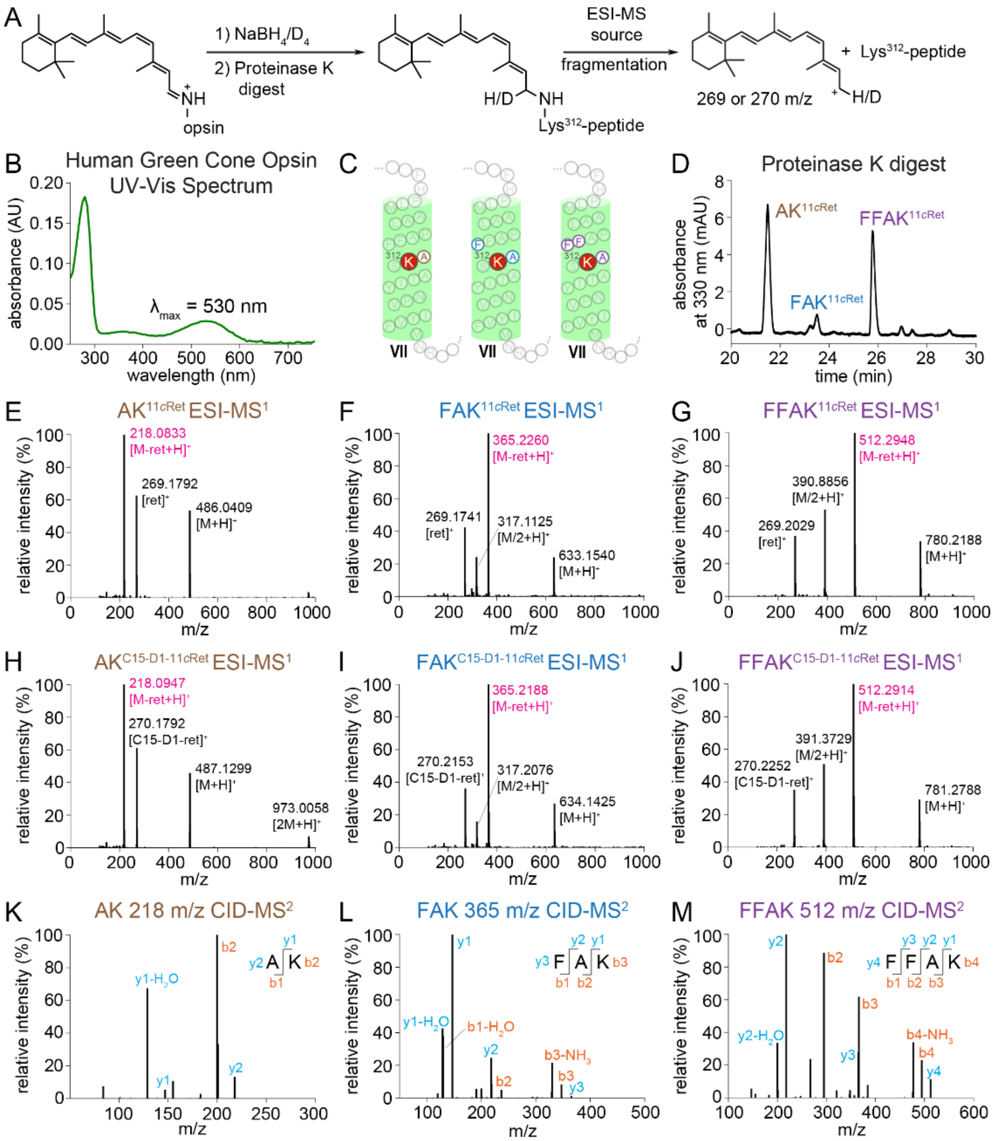

Figure S4. LC-MS/MS analysis of proteinase K digests of green-cone opsin purified in LMNG-detergent micelles. (A) Schematic diagram of sample preparation and MS workflow. (B) UV-Vis spectrum of purified green-cone opsin in LMNG. (C) Location of chromophore-binding residue within helix VII of green-cone opsin, with labeled N^{ε}-retinyl-peptide fragments detected from the proteinase K digest. (D) Chromatographic separation of N^{ε}-retinyl-peptides from proteinase K digestion of purified green-cone opsin, treated with $NaBH_4$ or $NaBD_4$ in *i*PrOH. (E-J) ESI-MS^1 spectra showing characteristic cleavage of the retinyl cation from precursor retinyl-peptide analyte, producing a product peptide peak. (K-M) MS^2 spectra of CID fragmentation of product peptide ion for sequence determination.

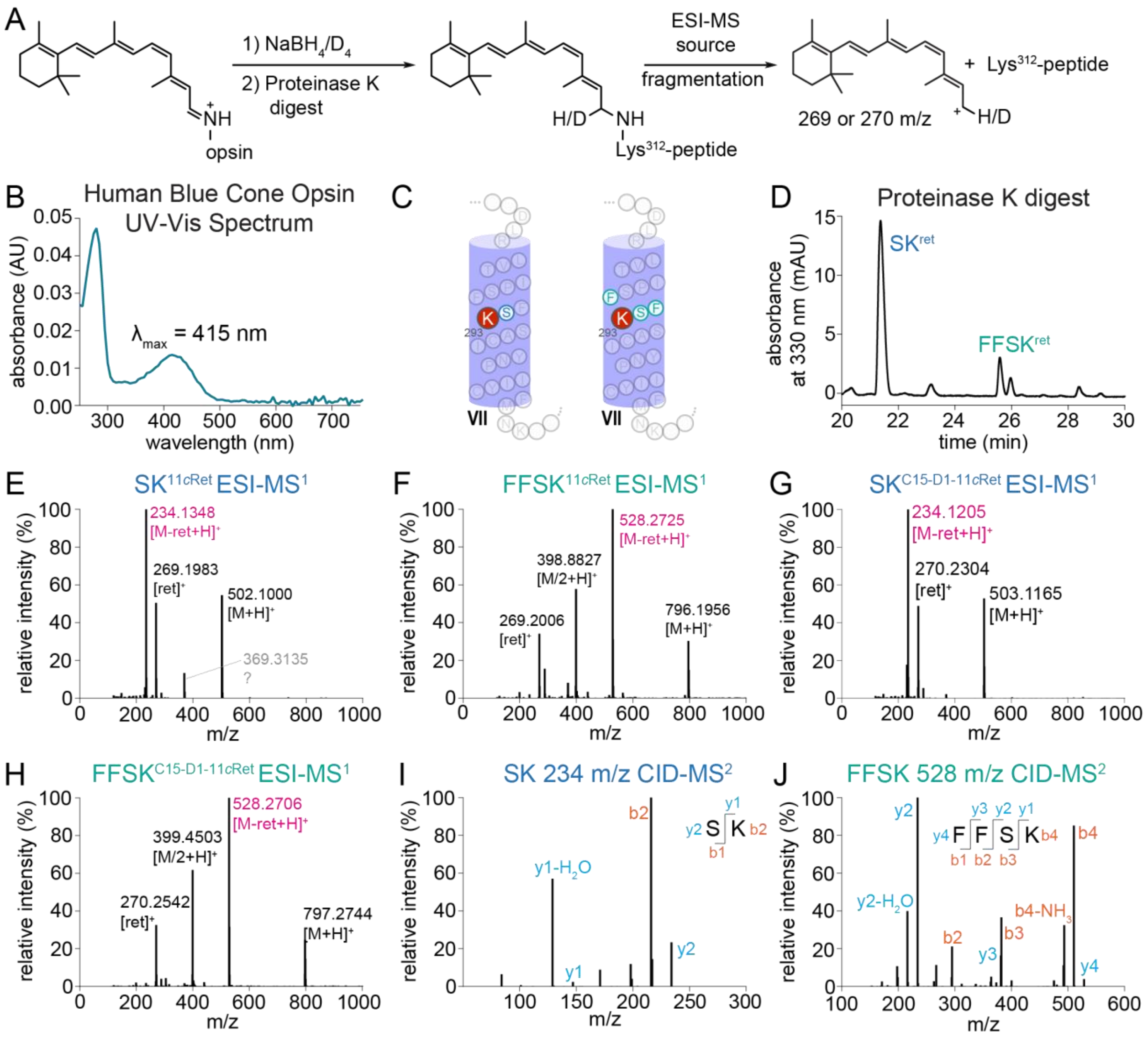

Figure S5. LC-MS/MS analysis of proteinase K digests of blue cone opsin purified in LMNG detergent micelles. (A) Schematic diagram of sample preparation and MS workflow. (B) UV-Vis spectrum of purified blue-cone opsin in LMNG. (C) Location of chromophore-binding residue within helix VII of blue-cone opsin, with labeled N^{ε}-retinyl-peptide fragments detected from the proteinase K digest. (D) Chromatographic separation of N^{ε}-retinyl-peptides from proteinase K digestion of purified blue-cone opsin, treated with $NaBH_4$ or $NaBD_4$ in *i*PrOH. (E-H) ESI-MS^1 spectra showing characteristic cleavage of the retinyl cation from precursor retinyl-peptide analytes, producing product peptide peaks. (I, J) MS^2 spectra of CID fragmentation of product peptide ions for sequence determination.

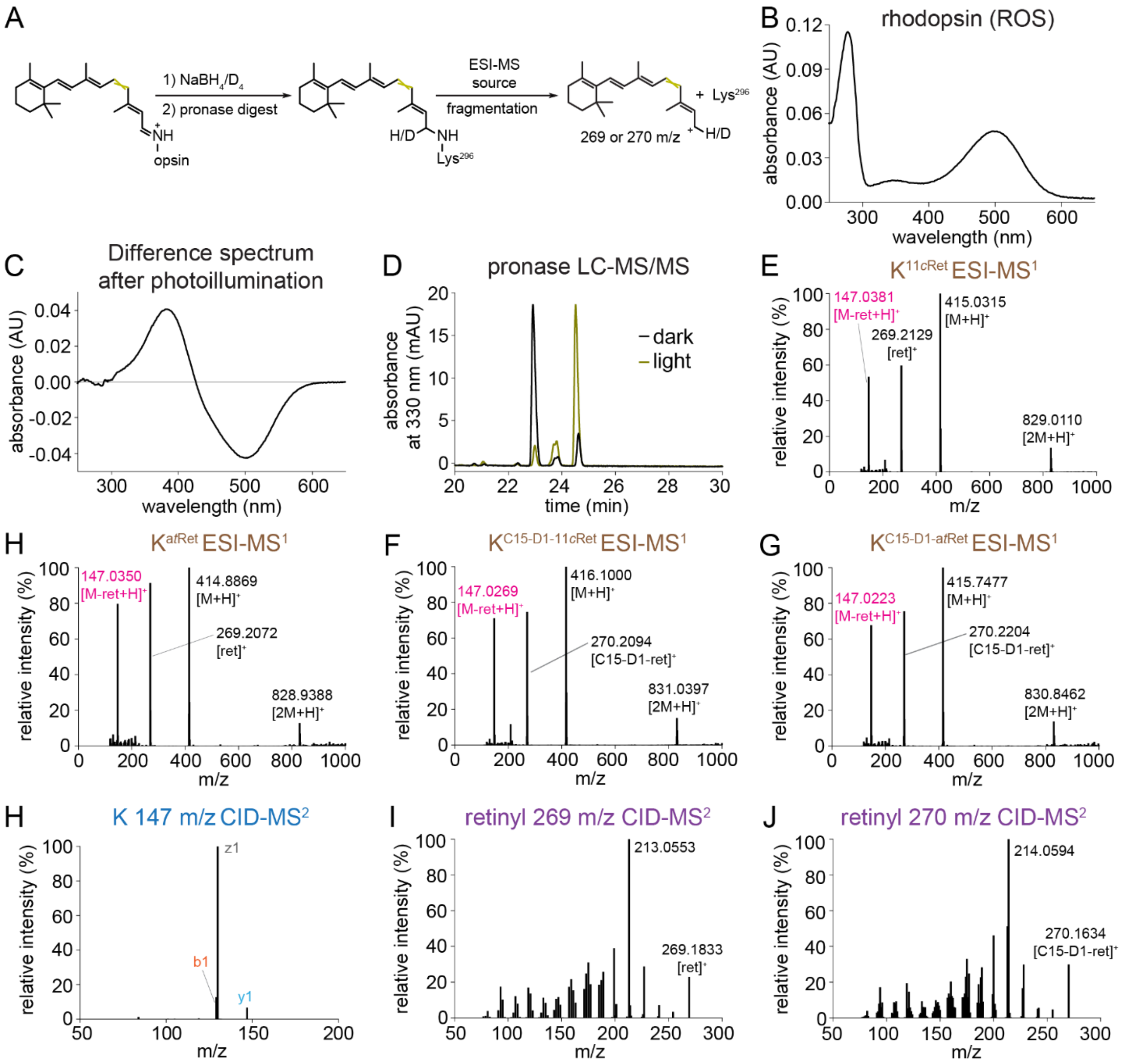

Figure S6. LC-MS/MS analysis of pronase digest of bRho from bROS membranes. (A) Schematic diagram of sample preparation and MS workflow. (B) UV-Vis spectrum of bRho in bROS membranes solubilized in LMNG. (C) Difference absorbance spectrum after illumination (subtracting the ground-state spectrum) of bROS membranes with 505-nm fiber light at 125 µW for 10 sec at 4 °C. (D) Chromatographic separation of N^{ε}-retinyl-Lys peaks from pronase digest of ground-state and photoactivated bRho treated with $NaBH_4$ or $NaBD_4$ in *i*PrOH. (E-H) ESI-MS^1 spectra showing characteristic cleavage of retinyl cation from precursor N^{ε}-retinyl-Lys analyte, producing a product Lys ion. (I) CID-MS^2 spectrum of the product Lys ion exhibiting the characteristic fragmentation pattern of Lys. (J, K) CID-MS^2 spectrum of retinyl cation displaying

complex fragmentation patterns with a characteristic dominant 213 m/z signal from $NaBH_4$-reduced bRho and 214 m/z signal for $NaBD_4$-reduced bRho (49,50).

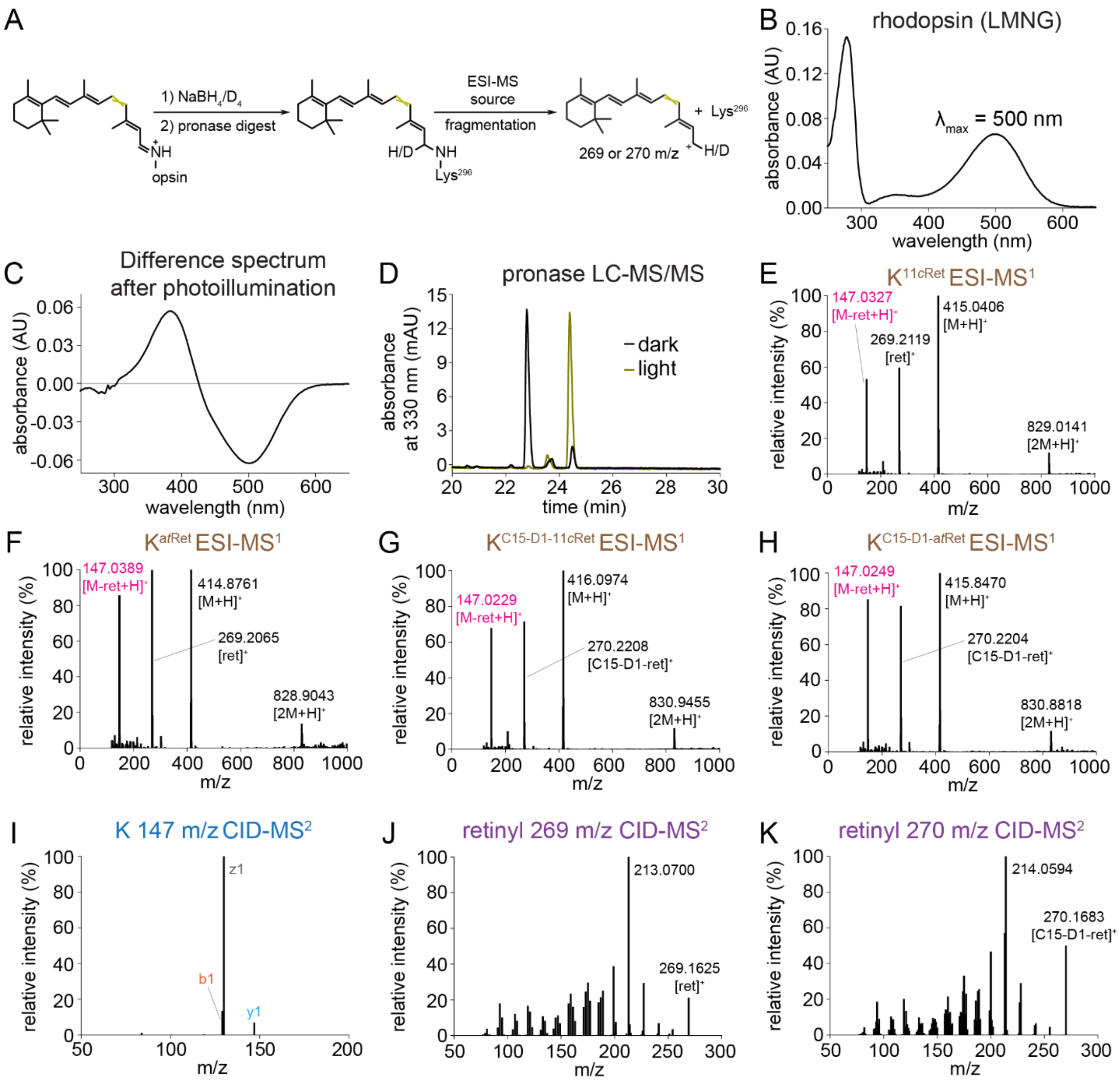

Figure S7. LC-MS/MS analysis of pronase digest of bRho purified in LMNG detergent. (A) Schematic diagram of sample preparation and MS workflow. (B) UV-Vis spectrum of purified bRho in LMNG. (C) Difference absorbance spectrum after illumination (post-illumination *minus* pre-illumination spectrum) of bRho with 505-nm fiber light at 125 µW for 10 sec at 4 °C. (D) Chromatographic separation of N^{ε}-retinyl-Lys peaks from pronase digest of ground-state and

photoactivated bRho, treated with $NaBH_4$ or $NaBD_4$ in *i*PrOH. (E-H) ESI-MS[1] spectra showing characteristic cleavage of the retinyl cation from precursor N^{ε}-retinyl-Lys analytes, producing a product Lys ion. (I) CID-MS[2] spectrum of the product Lys ion exhibiting the characteristic fragmentation pattern of Lys. (J, K) CID-MS[2] spectrum of retinyl cation demonstrating complex fragmentation pattern with characteristic dominant 213 m/z signal for $NaBH_4$-reduced bRho and 214 m/z signal for $NaBD_4$-reduced bRho (49,50).

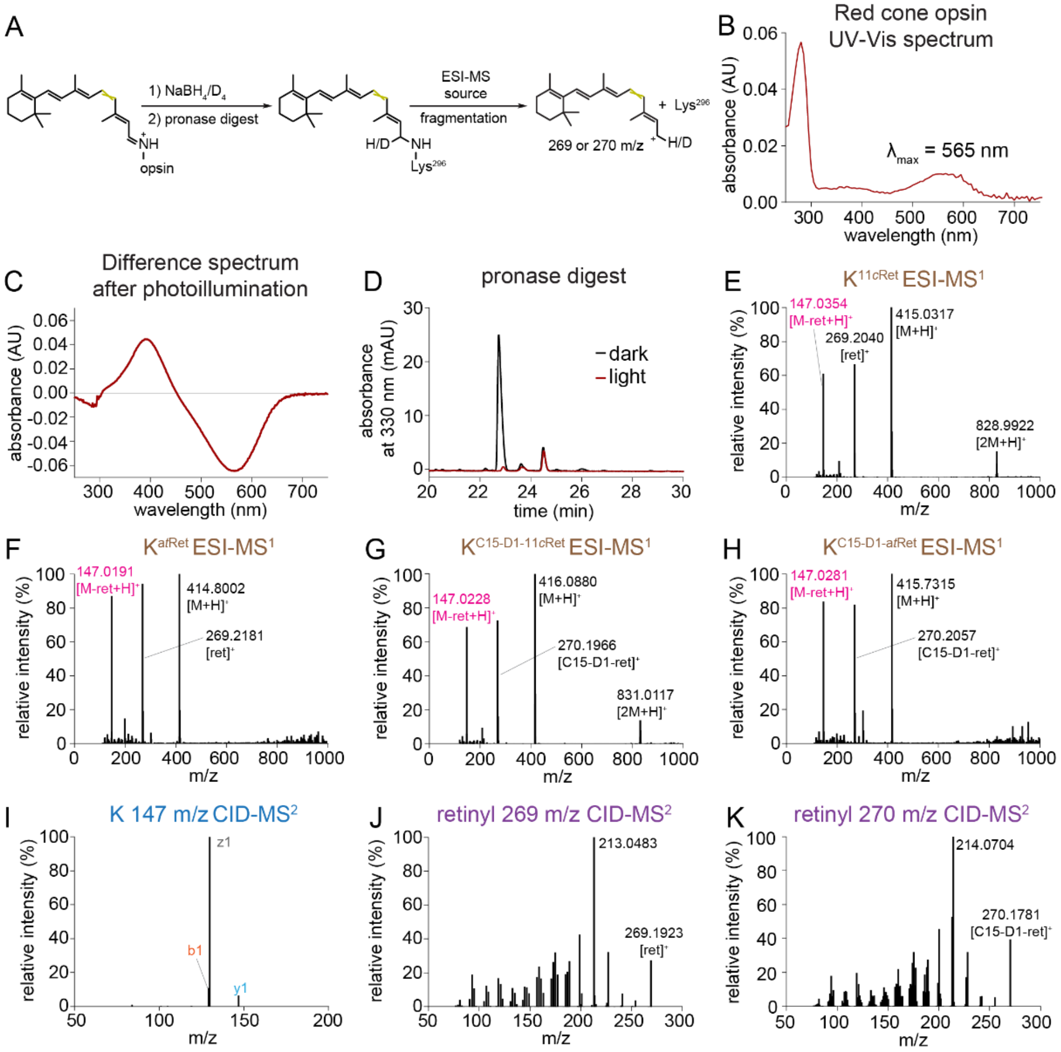

Figure S8. LC-MS/MS analysis of pronase digest of red cone opsin purified in LMNG detergent. (A) Schematic diagram of sample preparation and MS workflow. (B) UV-Vis spectrum of purified Red in LMNG. (C) Difference absorbance spectrum after illumination (subtracting the ground-state spectrum) of red cone opsin with 565-nm fiber light at 125 µW for 10 sec at 4 °C. (D) Chromatographic separation of N^{ε}-retinyl-Lys peaks from pronase digest of

ground-state and photoactivated red cone opsin, treated with $NaBH_4$ or $NaBD_4$ in *i*PrOH. (E-H) ESI-MS[1] spectra showing characteristic cleavage of retinyl cation from precursor N^{ε}-retinyl-Lys analyte, producing a product Lys ion. (I) CID-MS[2] spectrum of the product Lys ion exhibiting the characteristic fragmentation pattern of Lys. (J, K) CID-MS[2] spectrum of retinyl cation demonstrating complex fragmentation pattern with characteristic dominant 213 m/z signal for $NaBH_4$-reduced red-cone opsin and 214 m/z signal for $NaBD_4$-reduced red-cone opsin (49,50).

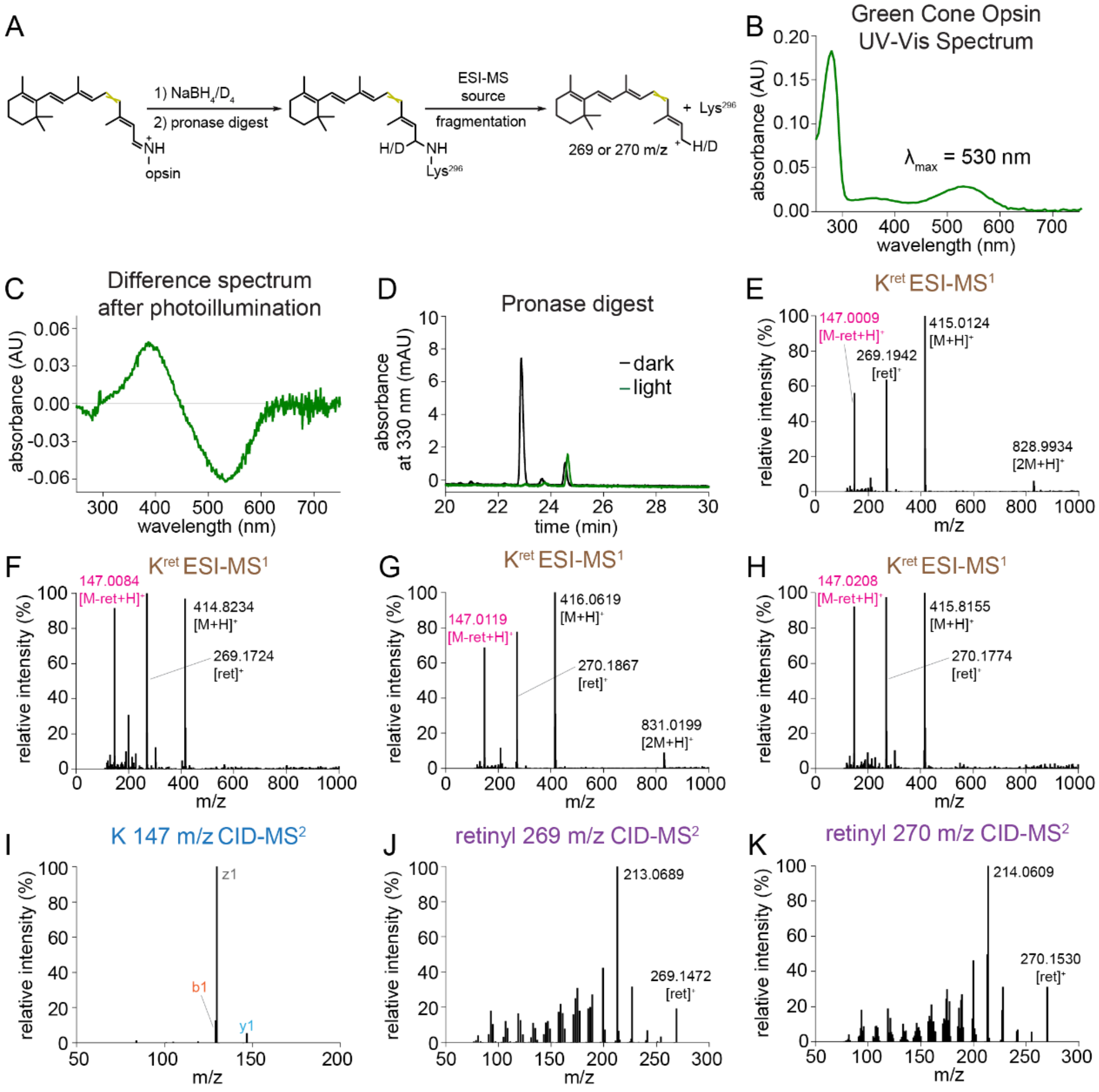

Figure S9. LC-MS/MS analysis of pronase digest of green cone opsin purified in LMNG detergent. (A) Schematic diagram of sample preparation and MS workflow. (B) UV-Vis spectrum of purified Green in LMNG. (C) Difference absorbance spectrum after illumination (post-illumination *minus* pre-illumination spectrum) of green cone opsin with 530-nm fiber light at 125 µW for 10 sec at 4 °C. (D) Chromatographic separation of N^{ε}-retinyl-Lys peaks from

pronase digest of ground-state and photoactivated green cone opsin, treated with $NaBH_4$ or $NaBD_4$ in *i*PrOH. (E, H) ESI-MS^1 spectrum showing characteristic cleavage of retinyl cation from precursor N^{ε}-retinyl-Lys analyte, producing a product Lys ion. (F, I) CID-MS^2 spectrum of the product Lys ion exhibiting the characteristic fragmentation pattern of Lys. (G, J) CID-MS^2 spectrum of the retinyl cation demonstrating complex fragmentation pattern with characteristic dominant 213 m/z signal for $NaBH_4$-reduced green-cone opsin and 214 m/z signal for $NaBD_4$-reduced green-cone opsin (49,50).

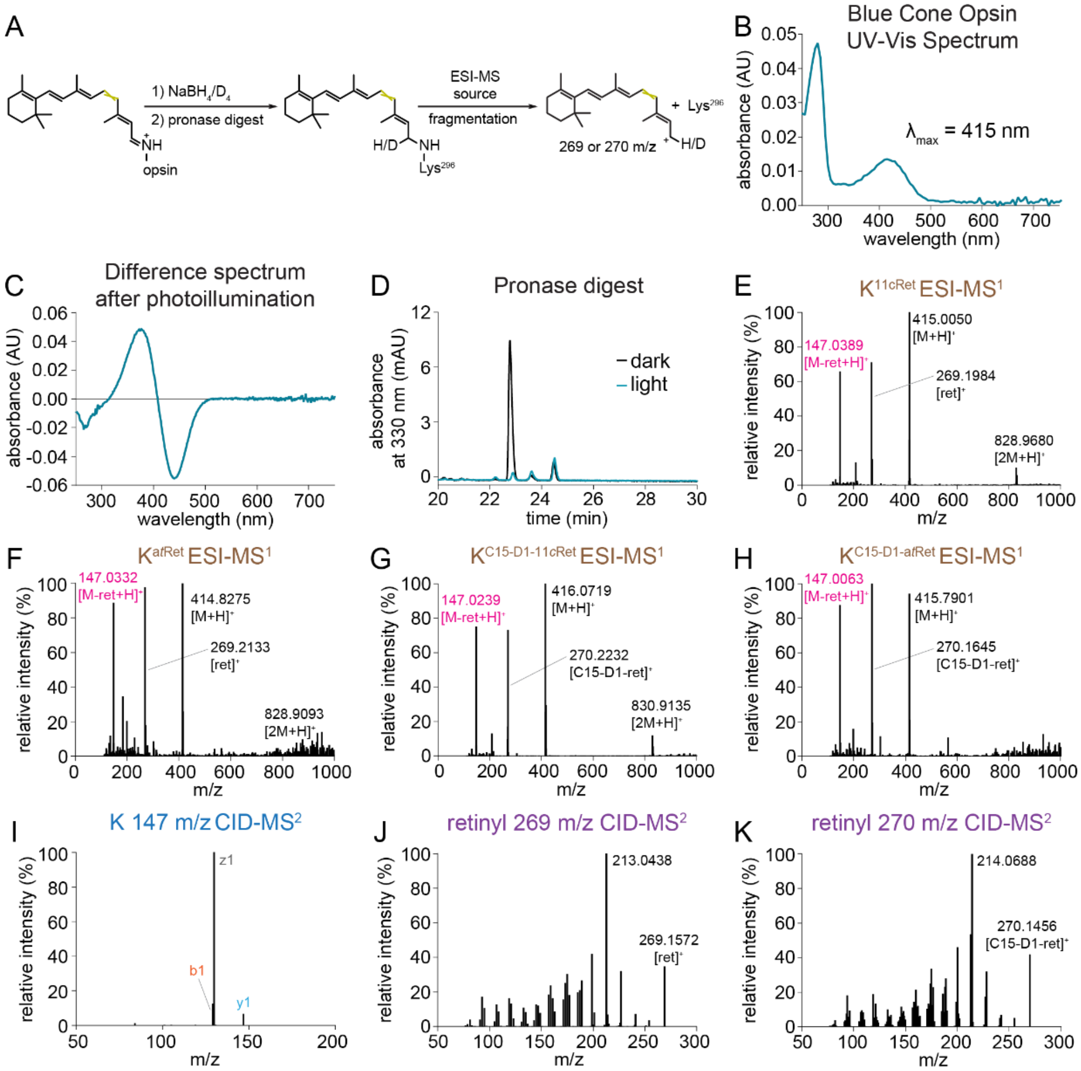

Figure S10. LC-MS/MS analysis of pronase digests of blue-cone opsin, purified in LMNG detergent (A) Schematic diagram of sample preparation and MS workflow. (B) UV-Vis spectrum of purified Blue in LMNG. (C) Difference absorbance spectrum after illumination (post-illumination *minus* pre-illumination spectrum) of blue cone opsin with 455-nm fiber light at 125 µW for 10 sec at 4 °C. (D) Chromatographic separation of N^{ε}-retinyl-Lys peaks from pronase

digest of ground-state and photoactivated blue cone opsin, treated with $NaBH_4$ or $NaBD_4$ in *i*PrOH. (E-H) ESI-MS[1] spectra showing characteristic cleavage of the retinyl cation from precursor N^{ε}-retinyl-Lys analytes, producing a product Lys ion. (I) CID-MS[2] spectrum of the product Lys ion, exhibiting the characteristic fragmentation pattern of Lys. (J, K) CID-MS[2] spectra of the respective retinyl cations, displaying complex fragmentation patterns with a characteristic dominant 213 m/z signal for $NaBH_4$-reduced blue-cone opsin, and a 214 m/z signal for $NaBD_4$-reduced blue-cone opsin (49,50).

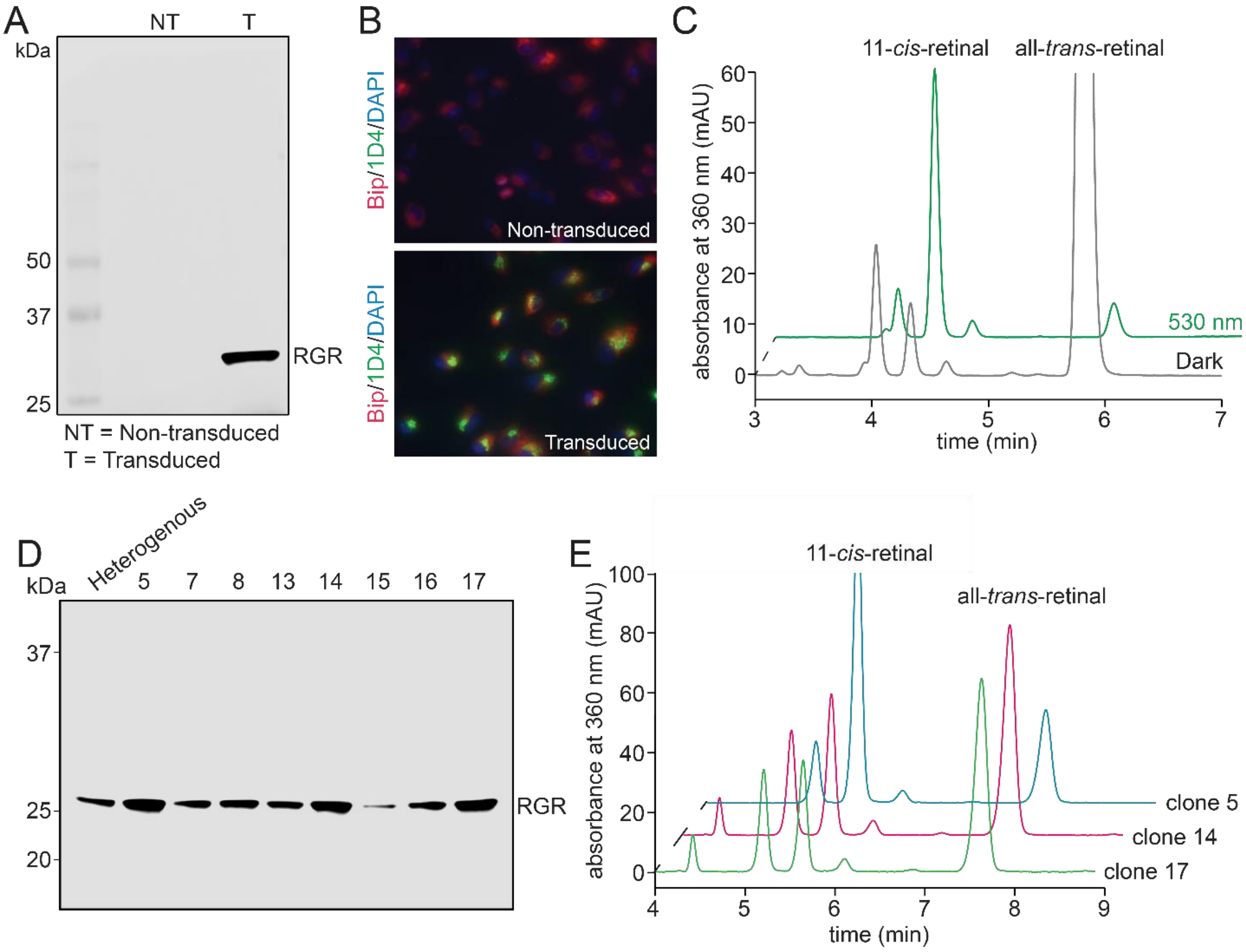

Figure S11. Generation of the bRGR HEK293 cell line. (A) Immunoblot of HEK293S and HEK293S-bRGR cells. (B) Subcellular localization of bRGR. The merged images show the colocalization of bRGR with the endoplasmic reticulum marker, BiP. (C) HPLC analysis of retinoids extracted from HEK293S-bRGR cells after addition of all-*trans*-retinal and either dark incubation or exposure to 530 nm light. (D) Immunoblot comparing relative expression of bRGR

in various clones of HEK293T-bRGR monoclonal cells. (E) HPLC analysis of retinoids extracted from HEK293S-bRGR monoclonal cells to compare photoisomerization activities.

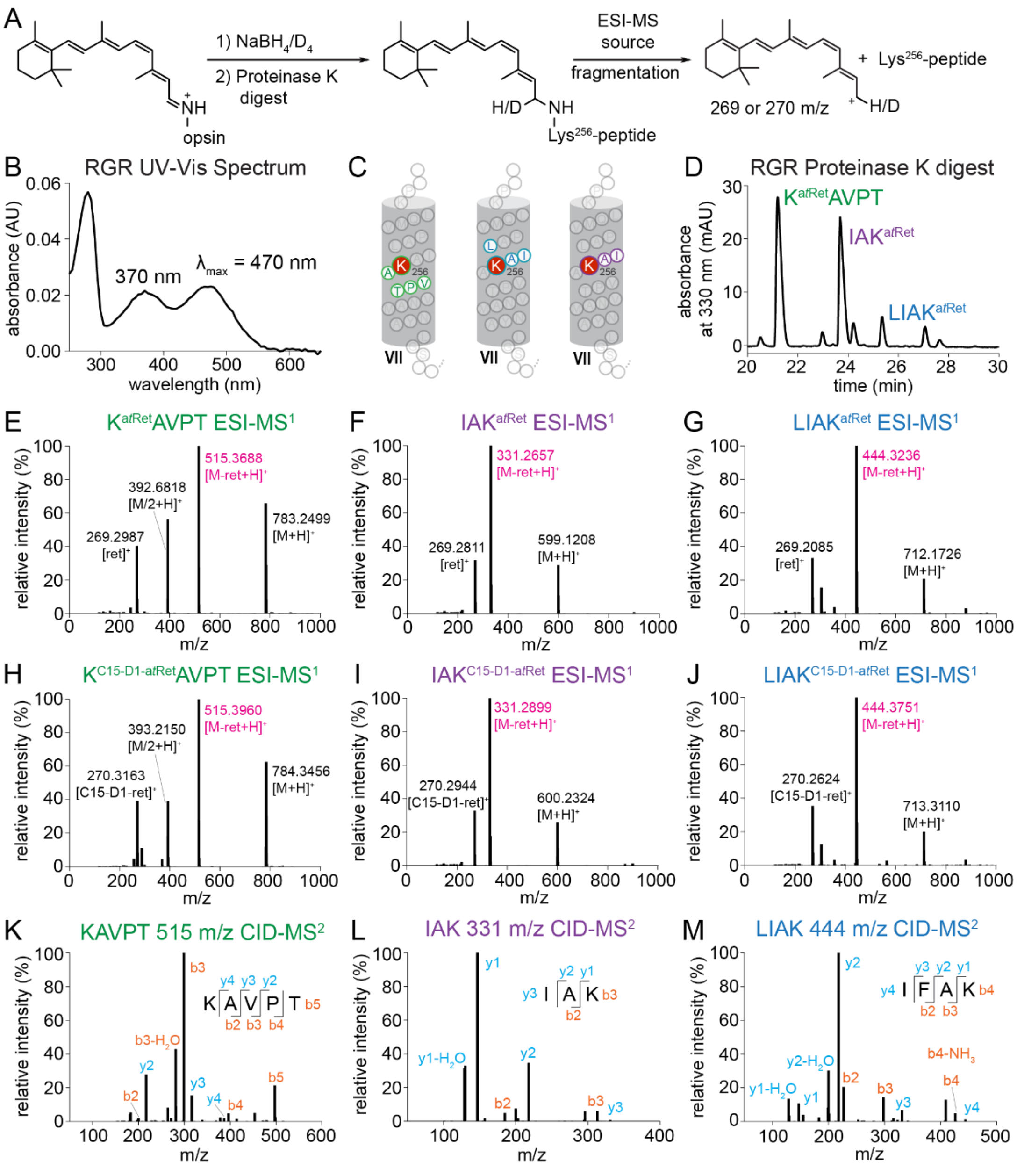

Figure S12. LC-MS/MS analysis of proteinase K digests of bRGR purified in LMNG detergent micelles. (A) Schematic diagram of sample preparation and MS workflow. (B) UV-Vis spectrum of recombinant bRGR reconstituted with all-*trans*-retinal and purified in LMNG. (C) Location of chromophore-binding residue within helix VII of bRGR, with labeled N^{ε}-retinyl-peptide fragments detected from the proteinase K digest. (D) Chromatographic separation of N^{ε}-

retinyl-peptides from proteinase K digestion of purified bRGR, treated with $NaBH_4$ or $NaBD_4$ in *i*PrOH. (E-J) ESI-MS[1] spectra showing characteristic cleavage of the retinyl cation from precursor retinyl-peptide analytes, producing product peptide peaks. (K-M) MS[2] spectra of CID fragmentation of product peptide ion for sequence determination.

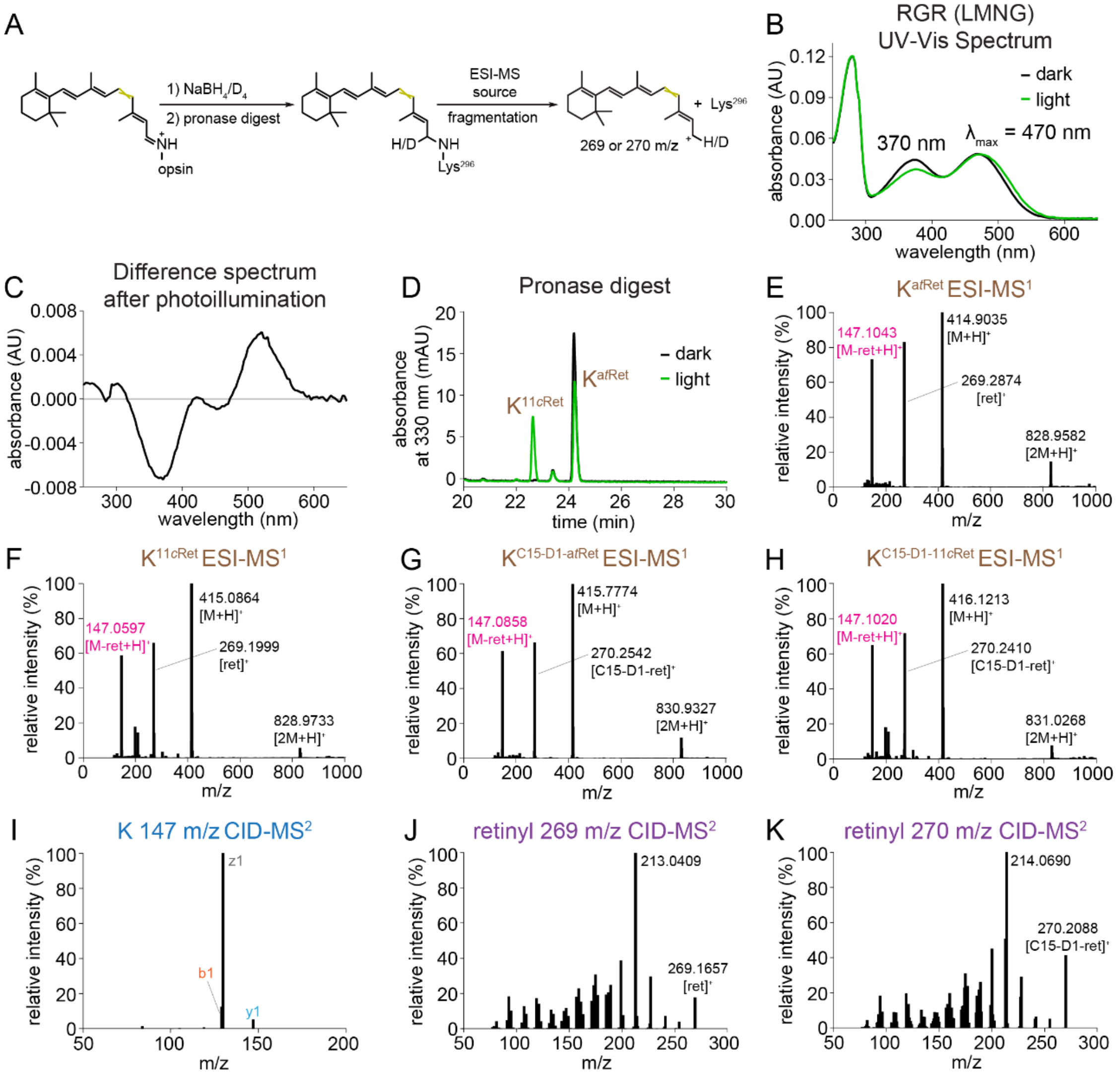

Figure S13. LC-MS/MS analysis of pronase digests of bRGR purified in LMNG detergent. (A) Schematic diagram of sample preparation and MS workflow. (B) UV-Vis spectrum of recombinant bRGR reconstituted with all-*trans*-retinal and purified in LMNG. (C) Difference absorbance spectrum after illumination (post-illumination *minus* pre-illumination spectrum) of bRGR with 530-nm fiber light at 125 µW for 10 sec at 4 °C. (D) Chromatographic separation of N^{ε}-retinyl-Lys peaks from pronase digest of bRGR before and after illumination, treated with

NaBH$_4$ or NaBD$_4$ in *i*PrOH. (E-H) ESI-MS[1] spectra showing the characteristic cleavage of the retinyl cation from precursor N$^{\varepsilon}$-retinyl-Lys analytes, producing a product Lys ion. (I) CID-MS[2] spectrum of the product Lys ion, exhibiting the characteristic fragmentation pattern of Lys. (J, K) CID-MS[2] spectra of the respective retinyl cations, displaying complex fragmentation patterns with a characteristic dominant 213 m/z signal for NaBH$_4$-reduced bRGR, and a 214 m/z signal for NaBD$_4$-reduced bRGR (49,50).

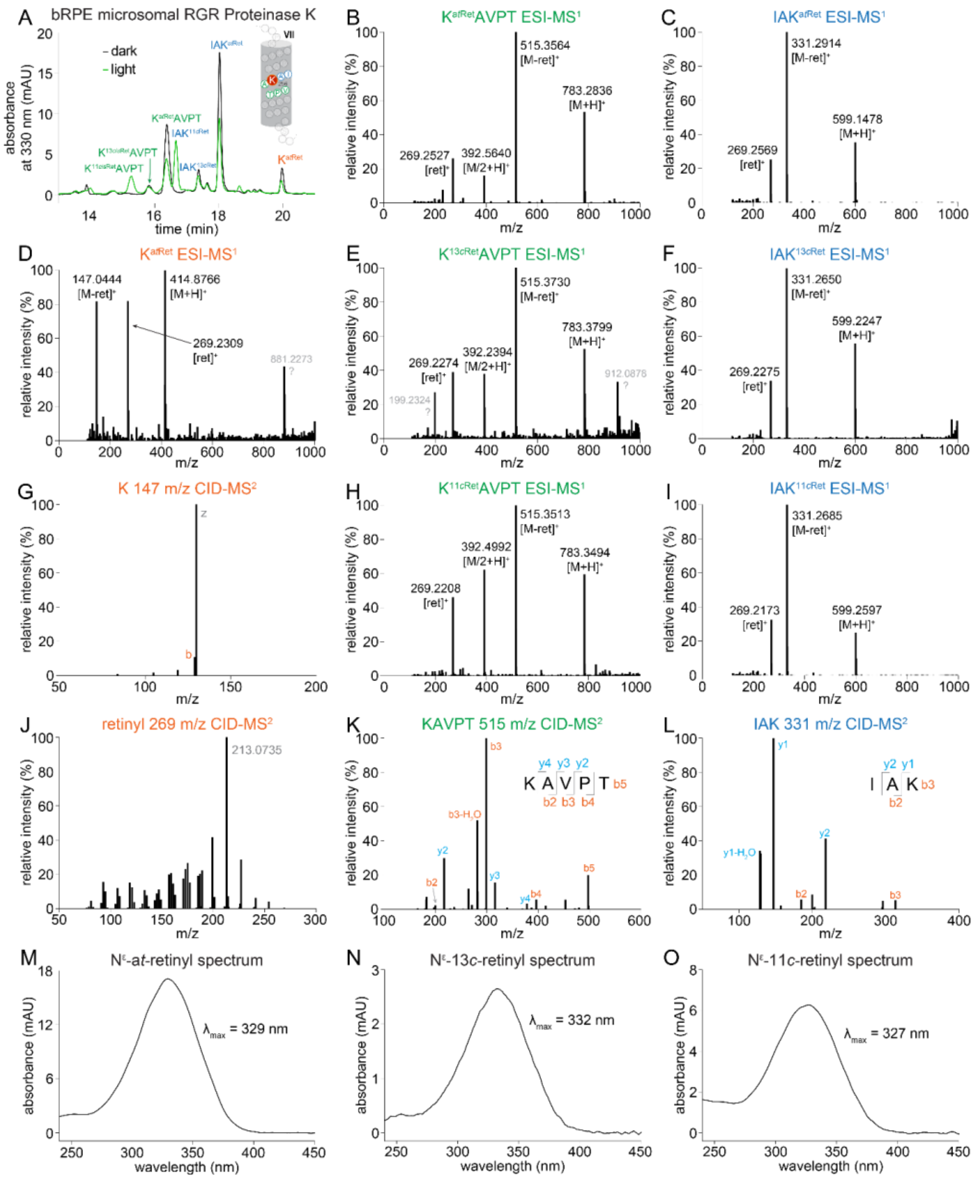

Figure S14. LC-MS/MS analysis of proteinase K digests of bRGR from bRPE microsomes. (A) Chromatographic separation of N$^{\varepsilon}$-retinyl-peptides from proteinase K digestion of purified

bRGR, treated with $NaBH_4$ or $NaBD_4$ in *i*PrOH. (B-F, H-I) ESI-MS[1] spectra showing characteristic cleavage of retinyl cation from precursor retinyl-peptide analyte, producing product peptide peaks. (G, K-L) MS[2] spectra of CID fragmentation of product peptide ion for sequence determination. (J) CID-MS[2] spectrum of retinyl cation demonstrating a complex fragmentation pattern with a characteristic dominant 213 m/z signal for $NaBH_4$-reduced bRGR, and a 214 m/z signal for $NaBD_4$-reduced bRGR (49,50). (M-O) UV-Vis absorption spectra collected during chromatography showing the characteristic spectrum for each retinyl-peptide isomer.

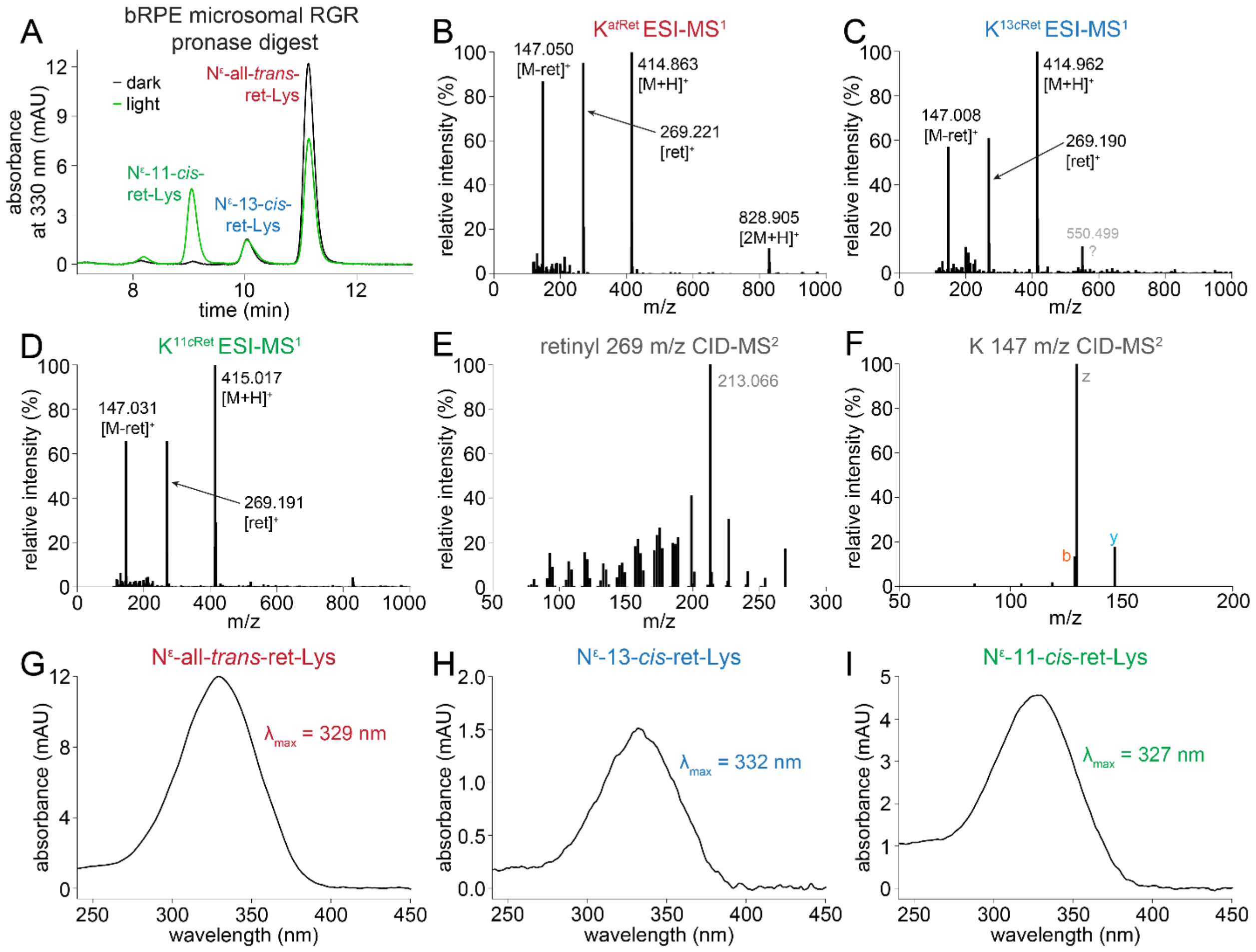

Figure S15. LC-MS/MS analysis of the pronase digest of bRGR from bRPE microsomes. (A) Chromatographic separation of N$^{\varepsilon}$-retinyl-Lys peaks from pronase digest of bRGR before and after illumination, treated with $NaBH_4$ or $NaBD_4$ in *i*PrOH. (B-D) ESI-MS[1] spectra showing characteristic cleavage of the retinyl cation from precursor N$^{\varepsilon}$-retinyl-Lys analyte, producing a product Lys ion. (E) CID-MS[2] spectrum of the retinyl cation demonstrating complex

fragmentation pattern with characteristic dominant 213 m/z signal for $NaBH_4$-reduced bRGR, and a 214 m/z signal for $NaBD_4$-reduced bRGR (49,50). (F) CID-MS^2 spectrum of the product Lys ion exhibiting the characteristic fragmentation pattern of Lys. (G-I) UV-Vis absorption spectra collected during chromatography, showing the characteristic spectrum for each retinyl-peptide isomer.

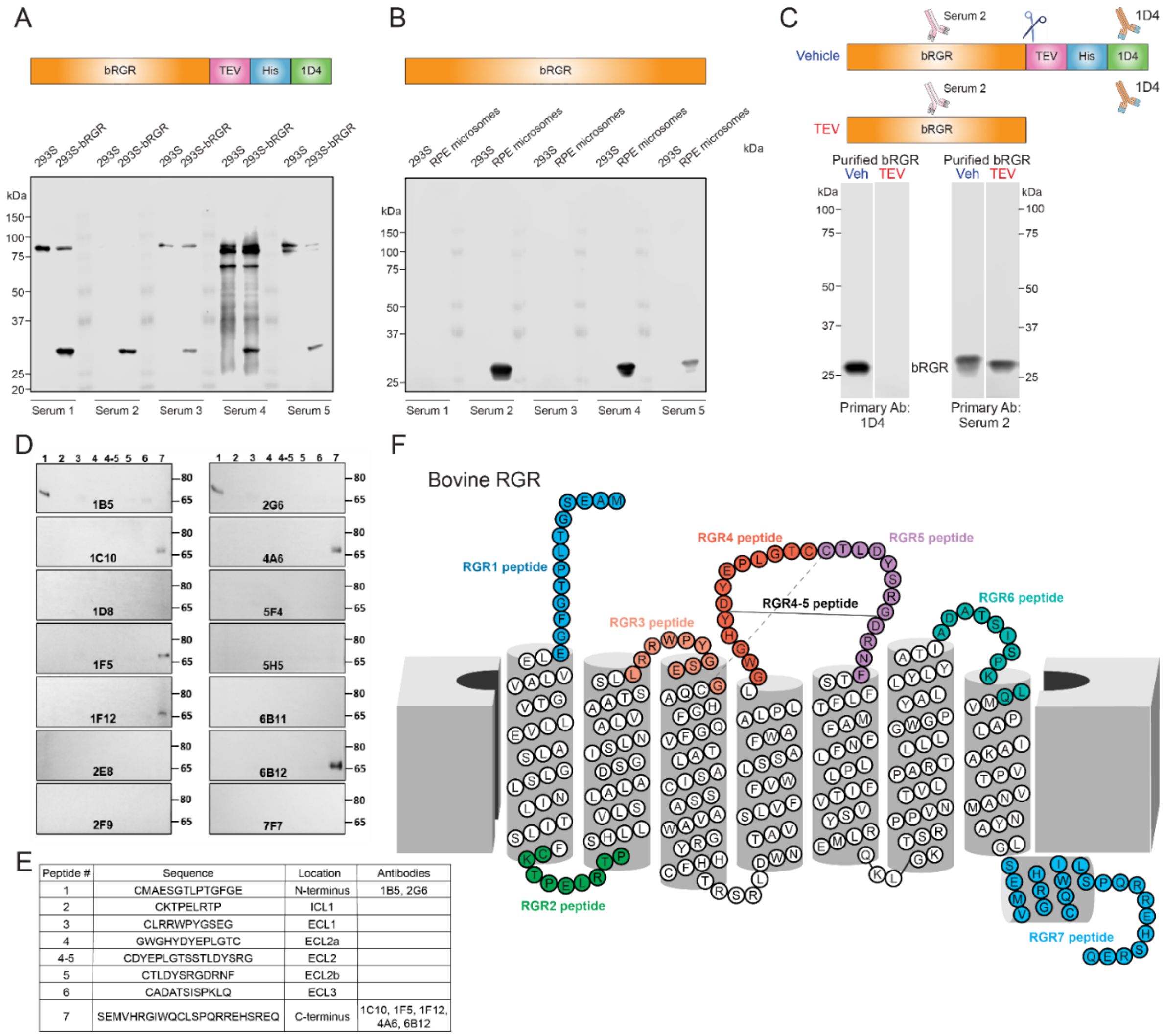

E

Peptide #	Sequence	Location	Antibodies
1	CMAESGTLPTGFGE	N-terminus	1B5, 2G6
2	CKTPELRTP	ICL1	
3	CLRRWPYGSEG	ECL1	
4	GWGHYDYEPLGTC	ECL2a	
4-5	CDYEPLGTSSTLDYSRG	ECL2	
5	CTLDYSRGDRNF	ECL2b	
6	CADATSISPKLQ	ECL3	
7	SEMVHRGIWQCLSPQRREHSREQ	C-terminus	1C10, 1F5, 1F12, 4A6, 6B12

Figure S16. Production of bRGR monoclonal antibodies. (A) Evaluation of sera from mice immunized with bRGR to assess antibody production against bRGR-TEV-His-1D4. Immunoblot analysis was performed using lysates from HEK293S and HEK293-bRGR cells. (B) Evaluation of sera from mice injected with bRGR to assess antibody production against bRGR. Immunoblot analysis was performed using lysates from HEK293S microsomes and bovine RPE microsomes. (C) Analysis of serum2 against purified bRGR-TEV-His-1D4 and purified bRGR following TEV cleavage to ensure antibody reactivity against bRGR. Serum 2 detected purified bRGR following TEV cleavage but the 1D4 antibody did not. (D) Screening of monoclonal hybridomas from the mouse that produced serum 2. Immunoblot analysis was performed using bovine RPE microsomes. (E) Peptides used to identify the epitope of antibodies produced by the selected monoclonal hybridoma. (F) Putative two-dimensional model of bRGR showing peptide sequences used for epitope mapping.

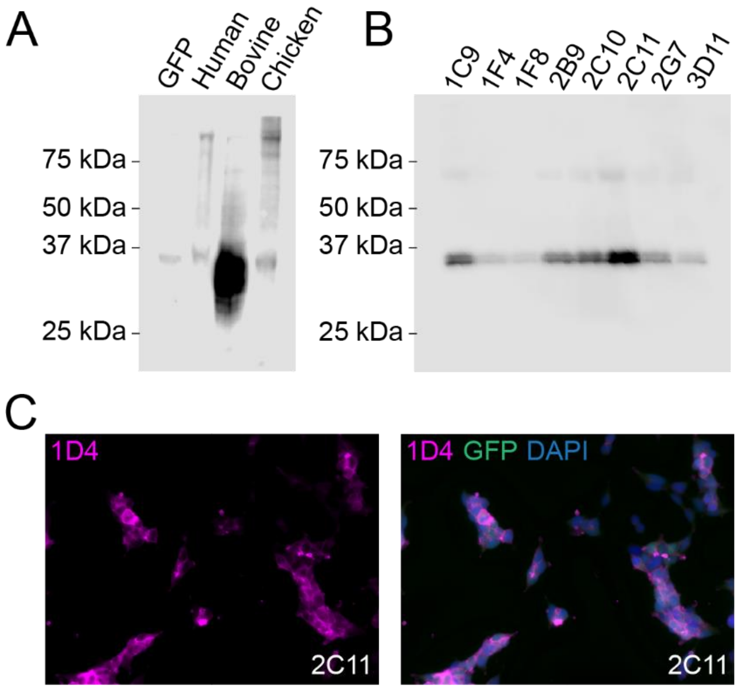

Figure S17. Generation of HEK293 cell line stably expressing bRRH. (A) Comparison by anti-1D4 western blot analysis of different species of bRRH, each with a C-terminal 1D4 tag transiently expressed in HEK293S cells. (B) Clonal selection of HEK293S cells stably expressing bRRH. (C) Immunostaining of HEK293S-bRRH cells against 1D4 tag and counterstained with DAPI. GFP is cotranslationally expressed.

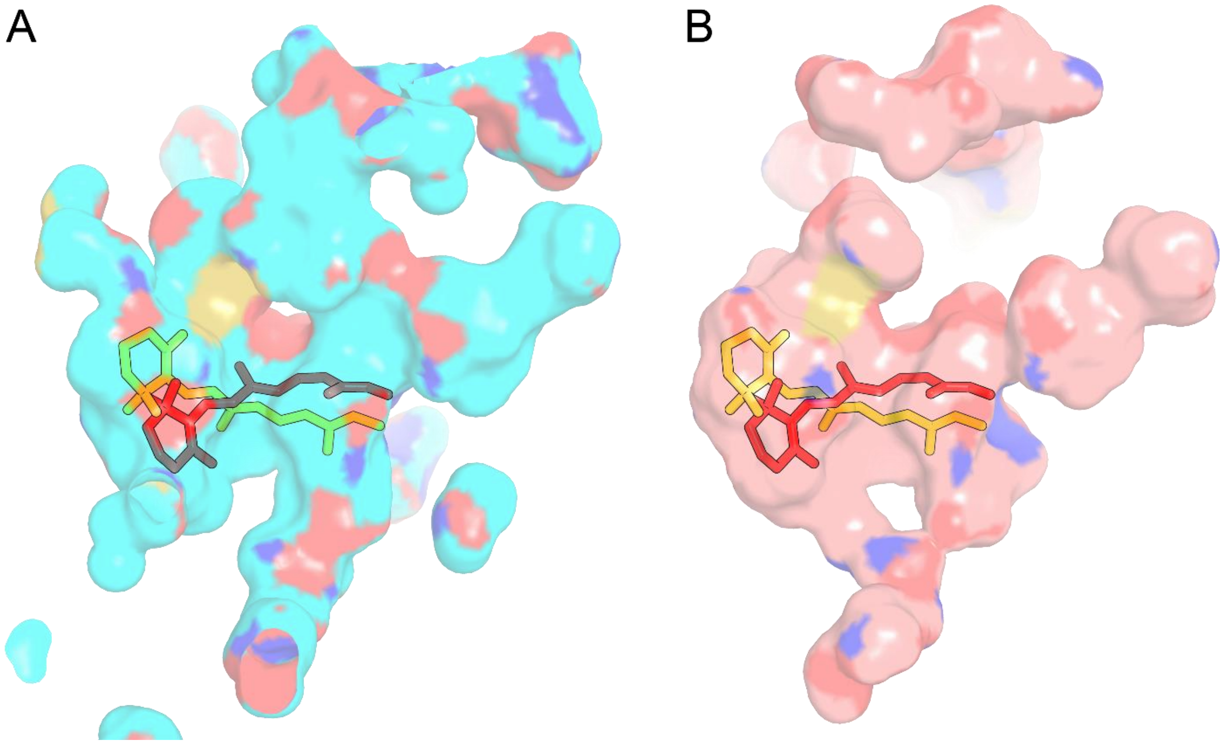

Figure S18. Comparison of the putative chromophore-binding pocket of vertebrate and invertebrate bRRH. AlphaFold models of bovine RRH (panel A) and *Hasarius adansoni's* RRH (panel B) are depicted in surface representation. The pocket alignment reveals that the binding pocket of bRRH is more accessible to water channels than that of HaRRH. 11-*cis*-retinal from bRho (red stick) and all-*trans*-retinal from bRho* (yellow stick) were included for reference.
